# Regulating Aggregation‐Induced Emission Luminogen for Multimodal Imaging‐Navigated Synergistic Therapy Involving Anti‐Angiogenesis

**DOI:** 10.1002/advs.202302713

**Published:** 2024-08-29

**Authors:** Fei Zhang, Jie Cui, Yao Zhang, Miao Yan, Xiaoxiao Wu, Xue Liu, Dingyuan Yan, Zhijun Zhang, Ting Han, Hui Tan, Dong Wang, Ben Zhong Tang

**Affiliations:** ^1^ Center for AIE Research Shenzhen Key Laboratory of Polymer Science and Technology Guangdong Research Center for Interfacial Engineering of Functional Materials College of Materials Science and Engineering Shenzhen University Shenzhen 518060 China; ^2^ Hubei Key Laboratory of Radiation Chemistry and Functional Materials School of Nuclear Technology and Chemistry & Biology Hubei University of Science and Technology Hubei 437000 China; ^3^ School of Health Service and Management Shanxi University of Chinese Medicine 121 University Street Jinzhong Shanxi 030619 China; ^4^ Department of Chemistry Xinzhou Normal University Xinzhou Shanxi 034000 China; ^5^ Xianning Public Inspection and Testing Center Xianning Hubei 437000 China; ^6^ Center for Child Care and Mental Health (CCCMH) Shenzhen Children's Hospital Shenzhen 518034 China; ^7^ School of Science and Engineering Shenzhen Institute of Aggregate Science and Technology The Chinese University of Hong Kong Shenzhen Guangdong 518172 China

**Keywords:** aggregation‐induced emission, anti‐angiogenesis, multimodal phototheranostics, NIR‐II FLI

## Abstract

As a new avenue for cancer research, phototheranostics has shown inexhaustible and vigorous vitality as it permits real‐time diagnosis and concurrent in situ therapy upon non‐invasive light‐initiation. However, construction of an advanced material, allowing prominent phototheranostic outputs and synchronously surmounting the inherent deficiency of phototheranostics, would be an appealing yet significantly challenging task. Herein, an aggregation‐induced emission (AIE)‐active luminogen (namely DBD‐TM) featured by intensive electron donor‐acceptor strength and twisted architecture with finely modulated intramolecular motion, is tactfully designed and prepared. DBD‐TM simultaneously possessed fluorescence emission in the second near‐infrared (NIR‐II) region and high‐efficiency photothermal conversion. By integrating DBD‐TM with anti‐angiogenic agent sorafenib, a versatile nanomaterial is smoothly fabricated and utilized for trimodal imaging‐navigated synergistic therapy involving photothermal therapy and anti‐angiogenesis toward cancer. This advanced approach is capable of affording accurate tumor diagnosis, complete tumor elimination, and largely restrained tumor recurrence, evidently denoting a prominent theranostic formula beyond phototheranostics. This study will offer a blueprint for exploiting a new generation of cancer theranostics.

## Graphical abstract

A versatile nanomaterial, comprised of a subtly regulated AIEgen and anti‐angiogenic agent, is constructed and actualized unprecedented performance on NIR‐II FLI‐PAI‐PTI trimodal imaging navigated synergistic PPT and anti‐angiogenesis therapy. This approach afforded accurate tumor diagnosis, complete tumor elimination, and largely restrained tumor recurrence, denoting a prominent theranostic formula beyond phototheranostics.

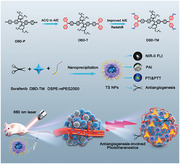

## 1 Introduction

Although numerous advances have been made in therapeutic efficiency, cancer is still a leading cause of death worldwide caused by high incidence and mortality. In view of the respective and collective drawbacks of conventional approaches, it is momentously significant to develop innovatory protocols for cancer treatment. Given the circumstances, phototheranostics^[^ ^1^ ^]^ that refers to the ingenious integration of diagnostic optical imaging and phototherapeutic interventions into a single formulation upon photoexcitation, is growing into a sparkling frontier in this area by virtue of its high selectivity in space and time, low toxicity and side effects, good efficacy, and prominent controllability and noninvasiveness.^[^ ^2^ ^]^ As one of phototherapeutic approaches,^[^ ^3^ ^]^ photothermal therapy (PTT)^[^ ^4^ ^]^ that refers to the utilization of light energy to produce localized hyperthermia for tumor ablation by using of photothermal conversion agents, has captivated much interest due to its high therapy efficiency comparing with photodynamic therapy (PDT), incidental photoacoustic imaging (PAI)/photothermal imaging (PTI) functions, as well as inherent characteristics as phototheranostics. Nevertheless, some obstacles restrain the practical applications of PTT. With hyperthermia, cytoprotective pathways could be activated to produce heat shock proteins (such as HSP70 and HSP90) and lead to serious thermoresistance, which would decrease the therapeutic effect.^[^ ^5^ ^]^ Moreover, relatively high temperature is usually required to achieve high‐efficiency therapeutics, but high temperature could induce both inflammation and the damage to surrounding normal tissues.^[^ ^6^ ^]^ Evidently, the development of synergistic therapeutic protocols is urgently needed to overcome the disadvantages of single PTT. Furthermore, it remains challenging to construct photothermal conversion agents featured by diagnostic fluorescence imaging (FLI) capability for implementing high‐performance phototheranostics, resulting from the profoundly competitive energy dissipation of radiative decay (FLI) and nonradiative decay (PTT, PAI and PTI). Therefore, it fundamentally calls for new theranostic system to integrate prominent phototheranostic behaviours originated from the well‐tailored agent with synergistic therapeutic modality, to approach super‐theranostics beyond phototheranostics.

Tumor blood vessels are nutrient channels by delivering oxygen and nutrients to tumors, which are major factors inducing tumor recurrence.^[^ ^7^ ^]^ Antiangiogenic therapy has been thus considered as one of innovatory methods for cancer treatment, by which the growth of tumors can be inhibited by impeding the formation of blood vessels in tumor areas, consequently cutting off the supply of nutrients.^[^ ^8^ ^]^ However, the single antiangiogenic therapy usually exhibits insufficient therapeutic outcomes.^[^ ^9^ ^]^ In the related context, the United States Food and Drug Administration proposal that anti‐angiogenesis techniques can be combined with other therapeutic strategies to achieve significant improvement in the survival rate of cancer patients.^[^ ^10^ ^]^ It is firmly believed the cooperation of anti‐angiogenesis and phototheranostics would be vitally important as a groundbreaking tactic to surmount respective drawbacks and approach synergistic effects with boosted cancer theranostics.

**Scheme 1:**
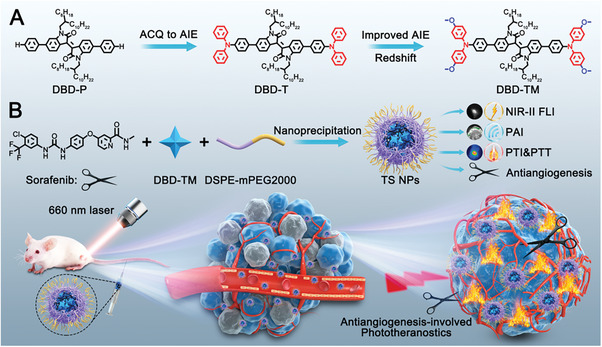
A) Chemical structures of DBD‐P, DBD‐T, and DBD‐TM. B) Nanofabrication of TS NPs and its application in integrated multimodal phototheranostics and antiangiogenesis.

Luminogens with aggregation‐induced emission (AIE) tendency have been recognized as an extraordinarily significant template to afford high‐performance multimodal phototheranostics by virtue of its distinctive capability on subtly regulating the balance between radiative and nonradiative energy dissipations by modulating intramolecular motions.^[^ ^11^ ^]^ In this contribution, we demonstrate for the first time the combination of multimodal phototheranostics on the basis of AIE luminogens (AIEgens) with anti‐angiogenesis. Molecular engineering was manipulated to achieve the transformation from conventional aggregation‐caused quenching (ACQ) to AIE effect, as well as the function elevations in terms of excitation/emission wavelength and photothermal conversion (**Scheme** **1**). On the basis of that, a versatile nanomaterial constituting by well‐tailored AIEgen DBD‐TM and anti‐angiogenic agent sorafenib was fabricated. In vitro and in vivo evaluations clearly manifested that the nanomaterial well performed in the second near‐infrared (NIR‐II) FLI‐PAI‐PTI trimodal imaging navigated synergistic PPT and anti‐angiogenesis therapy.

## 2 Results and Discussion

### 2.1 Molecular Design and Synthesis

Compounds, namely DBD‐P, DBD‐T and DBD‐TM, with different electron donator‐acceptor‐donator (D‐A‐D) configurations were rationally designed through connecting electron acceptor skeletons on both sides of (*E*)‐[3,3′‐biindolinylidene]−2,2′‐dione (isoindigo) unit, which has been widely utilized as a specialized electron acceptor moiety due to its relatively congested geometry and electron deficiency.^[^ ^12^ ^]^ Comparing with DBD‐P, the presence of biphenyl amine unit in DBD‐T can theoretically offer twisted conformation with molecular rotors, and thus enlarge the intermolecular distances, essentially preventing emission quenching in its aggregate state caused by intermolecular π‐π interaction. Moreover, the introduction of biphenyl amine group is able to endow DBD‐T with much larger D‐A interaction intensity and consequently reach remarkably red‐shifted excitation/emission wavelengths. In the case of DBD‐TM, the addition of methoxy groups would continuously red‐shift the excitation/emission wavelengths and finally reach high‐performance phototheranostic outputs including diminished photoscattering, negligible autofluorescence, deeper detection depth and minimized photodamage. The synthesis routes of these compounds are relatively simple, and provide relatively high yields (Scheme S1; Figures S1–S9, Supporting Information).

### 2.2 Theoretical Calculations and Photophysical Properties

In order to further understand the conformation and electron cloud density distribution of DBD‐P, DBD‐T, and DBD‐TM in the ground state (S_0_), the density functional theory methods were conducted at the level of B3LYP/6‐31g(d). As shown in Figure S10 (Supporting Information), these compounds have similar chemical structures, with a proper dihedral angle (more than 30°) between triphenylamine and isoindigo, showing a distorted spatial configuration. The electron cloud maps of the highest occupied molecular orbital are mainly located on the whole molecules, but the electron cloud maps of the lowest unoccupied molecular orbital are distributed on the electron‐deficient isoindigo, showing evident intramolecular charge transfer (ICT) property.

**Figure 1:**
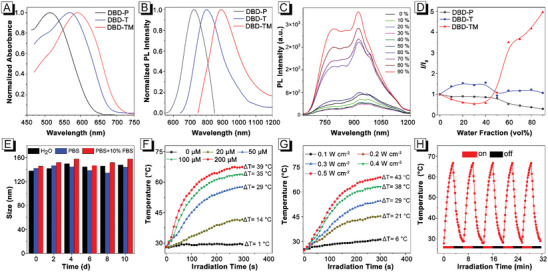
A) Normalized absorption spectra of DBD‐P, DBD‐T, and DBD‐TM (1.0 × 10^−5^ m) in THF. B) Normalized PL spectra in the solid state. C) PL spectra of DBD‐TM (1.0 × 10^−6^ m, λ_ex_: 592 nm) in THF solution with different water fractions. D) Plots of relative PL intensity (*I*/*I*_0_) in water/THF of DBD‐P, DBD‐T, and DBD‐TM. E) Stability analysis for size variation of TS NPs at a concentration of 100 µm measured by DLS at room temperature. F) Photothermal conversion of TS NPs (660 nm, 300 mW cm^−2^) in PBS at different concentrations. G) Photothermal conversion of TS NPs (100 µm, 300 mW cm^−2^) in PBS at different power densities. H) Photothermal stability of TS NPs after five on/off exposures in PBS (200 µm) with a 660 nm laser at a power density of 0.5 W cm^−2^.

The absorption spectra of DBD‐P, DBD‐T, and DBD‐TM were measured in tetrahydrofuran (THF). As shown in **Figure** **1**A, the absorption peaks of DBD‐P, DBD‐T, and DBD‐TM in THF were centered at 513, 572, and 598 nm, respectively, which was in consistence with the calculated gap values of Δ*E* (Figure S10, Supporting Information). In particular, DBD‐TM has a strong absorption at 660 nm, with the molar absorption coefficients (*ɛ*) of 1.9 × 10^4^ m ^−1^ cm^−1^ (Figure S11, Supporting Information). Subsequently, we determined the solid‐state fluorescence of those compounds. It was found that their maximum emission wavelengths were located at 727, 801, and 890 nm for DBD‐P, DBD‐T, and DBD‐TM respectively, and compound DBD‐TM also had strong emission in NIR‐II. The gradual redshift of emission wavelength is associated with the gradual enhancement of D‐A interaction, which is consistent with the experimental design expectation. To evaluate the AIE properties of these compounds, the PL spectra in THF with different fractions of water (*f_w_*) were measured. As illustrated in Figure 1C and Figure S12 (Supporting Information), DBD‐P is a typical ACQ molecule, while both DBD‐T and DBD‐TM have AIE performance. For DBD‐TM, in the system with low water content (*f*_w_ < 40%), the fluorescence intensity showed a decreased trend with the increasing of *f_w_*. This phenomenon was most likely caused by the twisted intramolecular charge transfer (TICT) of this compound, which was further proved by measuring its solvatochromism (Figure S13, Supporting Information), with the increase of the polarity solvent, the fluorescence emission gradually decreases, and the maximum emission wavelength is red‐shifted. When the *f_w_* in this system was more than 50%, its fluorescence intensity enhanced with the increased of *f_w_*, which was caused by the formation of aggregates that limited its intramolecular motions, indicating its typical AIE features. Moreover, the enhancement ratio of DBD‐TM was greater than that of DBD‐T (Figure 1D). Encouraged by their AIE tendency and long emission tailing in NIR‐II region, DBD‐T and DBD‐TM were further assessed. The quantum yield (QYs) of DBD‐T and DBD‐TM were determined to be 0.246% and 0.063% in THF, using ICG as a reference (Figure S14, Supporting Information). In order to make the hydrophobic AIE compounds have better dispersibility in water, DBD‐T and DBD‐TM nanoparticles (NPs) were prepared by precipitation methods using amphiphilic copolymer DSPE‐mPEG_2000_ as coating matrix. They respectively exhibited the QYs of 0.323% and 0.416% (Figure S15, Supporting Information), which were higher than those in solution state, solidly verifying the AIE behaviors.

### 2.3 Study on the Photothermal Properties

Subsequently, the photothermal conversion efficiency of DBD‐T and DBD‐TM was evaluated upon 660 nm laser irradiation with a power density of 0.3 W cm^−2^ in DMSO. As depicted in Figures S16 and S17 (Supporting Information), with the increasing of AIEgen concentration or power density, the temperature gradually enhanced, and its maximum temperature can be increased by 60–70 °C, indicating that it has good photothermal properties and shows potential application in phototheranostics. In addition, the photothermal conversion ability of DBD‐TM is superior to DBD‐T regardless of the change of concentration or power density. Notably, the photothermal conversion effect of both DBD‐T and DBD‐TM reserved subtle change after five continuous heating/cooling cycles upon irradiation by 660 nm laser (Figure 1H), indicating the high photostability, which is one of the essential peculiarities of prominent phototheranostic agent. As all discussed above, DBD‐TM shows long wavelength absorption in the NIR region, high molar absorption coefficients at 660 nm, good AIE performance, efficient photothermal conversion ability, and excellent thermal stability (Table S1, Supporting Information), which makes it have potential advantages in imaging and tumor therapy.

Sorafenib, an angiogenesis inhibitor, has been widely used in biomedicine.^[^ ^13^ ^]^ It is a hydrophobic small molecule without tumor‐targeting ability. Through the precipitation method, hydrophobic DBD‐TM and sorafenib were encapsulated with amphiphilic copolymer DSPE‐mPEG_2000_, and DBD‐TM@sorafenib NPs (TS NPs) with good dispersion in water were prepared. The encapsulation rates of DBD‐TM and sorafenib were 89.4% and 94.3% respectively by absorbance and HPLC‐MS analysis (Figure S18, Supporting Information). The size of the TS NPs was measured by dynamic light scattering (DLS), and the result showed that the average hydrodynamic diameter was ≈ 136 nm (Figure S19A, Supporting Information). Meanwhile, the transmission electron microscopy (TEM) measurements revealed that the size of the nanoparticles was ≈ 100–200 nm (Figure S19B, Supporting Information), and the morphology of the TS NPs were spherical, indicating the potential enhanced permeability and retention (EPR) effect in tumors. In addition, the nanoparticles remained uniformly dispersed after being placed in PBS for 10 days, manifesting the good dispersibility (Figure S19C, Supporting Information). DLS measurements indicated that the TS NPs had good stability in aqueous phase, PBS or 10% fetal bovine serum (Figure 1E). The maximum absorption peak of the TS NPs in PBS was 596 nm, and their emission spectra were located in 700–1200 nm (Figure S20, Supporting Information), elaborately revealing the potential application value in the fluorescence imaging of NIR‐II. The ability of TS NPs in water to convert NIR light energy into heat exposed to 660 nm laser was also evaluated. The thermal conversion capacity of TS NPs at different concentrations in water phase was measured at the power density of 0.3 W cm^−2^. The result showed that the thermal conversion temperature gradually elevated with the increasing of concentration (Figure 1F). At the same time, the thermal conversion capacity under different powers was measured at the concentration of 100 µm. It was found that the heat conversion temperature was also elevated gradually with the power density of lasers increased (Figure 1G). In particular, the near‐infrared thermal images were recorded at a concentration of 100 µm with a power of 0.3 W cm^−2^, which showed that the maximum temperature could reach 62.5 °C (Figure S21, Supporting Information). In addition, these nanoparticles can still reach the initial maximum temperature after five cycles, showing the exceptional photothermal stability (Figure 1H).

### 2.4 In Vitro Synergistic Therapy

**Figure 2:**
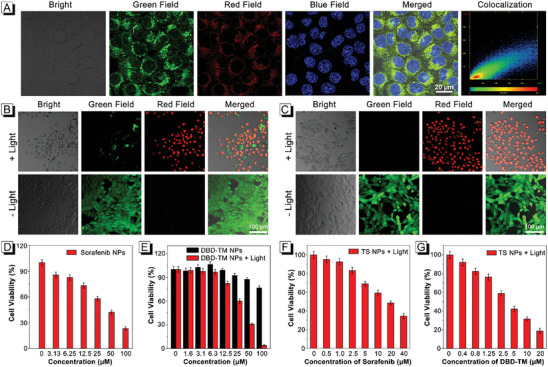
A) The CLSM images of 4T1 cells after treated with FITC@TS NPs and other dyes. Blue: Hoechst 33 342 (λ_ex_: 405 nm, emission filter: 425–465 nm); red: Lyso Tracker Deep Red (λ_ex_: 643 nm, emission filter: 660–700 nm); green: FITC@TS NPs (λ_ex_: 488 nm, emission filter: 510–600 nm). Live/dead CLSM images of 4T1 cells after treatment with B) 50 µg mL^−1^ and C) 100 µg mL^−1^ of DBD‐TM NPs with (up) or without (down) laser irradiation (laser: 660 nm, 0.3 W cm^−2^ for 5 min). The green field from Calcein‐AM is identified as live cells and red field from PI identified as dead cells. D) Viabilities of 4T1 cells after incubation with different concentrations of sorafenib NPs. E) Viabilities of 4T1 cells with DBD‐TM NPs with or without laser irradiation. F) Cells incubated with TS NPs with laser irradiation, and viabilities of 4T1 cells at different concentrations of sorafenib. G) Cells incubated with TS NPs with laser irradiation, and the viabilities of 4T1 cells at different concentrations of DBD‐TM.

In order to observe the distribution of TS NPs in cells, sorafenib, and DBD‐TM were encapsulated with fluorescein isothiocyanate labeled amphiphilic co‐polymer (DSPE‐mPEG_2000_‐FITC) providing FITC@TS NPs, which were then co‐incubated with commercial Lyso Tracker Deep Red dyes in the cells. The fluorescence confocal microscope observation showed that the green fluorescence of FITC in the FITC@TS NPs had good overlap with the red fluorescence of commercial Lyso Tracker Deep Red dye (**Figure** **2**A), and the Pearson's correlation coefficients were 82%, further indicated that FITC@TS NPs can target lysosomes through cellular endocytosis. The cytotoxicity of those nanomaterials on 4T1 cells was studied by MTT assay and live‐dead cell staining analysis. First, in the presence of sorafenib NPs, the cytotoxicity evaluation outputs demonstrated that the survival rate of cells gradually declined with the increase of sorafenib NPs concentration (Figure 2D), and the values of IC_50_ were calculated to be 37.68 µm, showcasing that the sorafenib could be used as a drug for tumor killing. In the case of DBD‐TM NPs, lower cytotoxicity was observed in the dark and suggested the good biocompatibility, while concentration‐dependent cytotoxicity was appeared upon laser irradiation (660 nm, 0.3 W cm^−2^, 5 min) (Figure 2E), and the value of IC_50_ was 45.08 µm, revealing its potential application value in tumor killing. Subsequently, the Calcein‐AM/PI staining experiment on cells was carried out. As shown in the Figure 2B, for DBD‐TM NPs, at a concentration of 50 µg mL^−1^, almost no apoptotic cell was present under dark conditions, but under laser irradiation (660 nm, 0.3 W cm^−2^, 5 min), a large number of apoptotic cells appeared and the proportion of living cells decreased. However, when the concentration reached 100 µg mL^−1^, apoptotic cells appeared under dark conditions, but almost all cells were apoptotic under the same laser irradiation (Figure 2C), further verifying the effect of phototherapeutics. Finally, the concentration‐dependent cytotoxicity of TS NPs was systematically studied. The experimental results exhibited that the values of IC_50_ for sorafenib (Figure 2F) and DBD‐TM (Figure 2G) contained in TS NPs under laser irradiation (660 nm, 0.3 W cm^−2^, 5 min) were calculated to be 18.18 and 3.61 µm, respectively, which were much lower than that in their independent existence. These results indicated that the single sorafenib NPs or DBD‐TM NPs cannot realize satisfactory cell‐killing efficacy. In addition, the combined index (CI) of sorafenib and DBD‐TM contained in TS NPs was 0.56, indicating that the combination of sorafenib and DBD‐TM had a synergistic effect on killing cancer cells (Table S2, Supporting Information).^[^ ^13^ ^]^ These experimental results showed that the significant improvement in the killing ability of TS NPs to cancer cells was achieved through the synergistic treatment of sorafenib and DBD‐TM, and subsequent in vivo studies were conducted.

### 2.5 In Vivo Imaging and Synergistic Therapy

**Figure 3:**
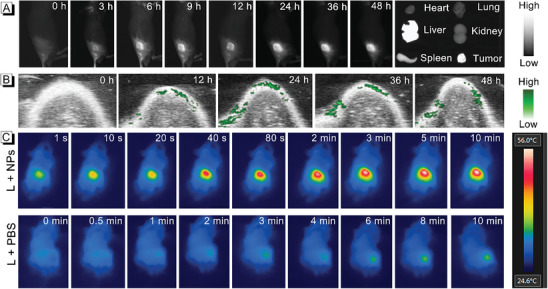
A) NIR‐II FLI and B) PAI of tumor area at different times after intravenous injection of TS NPs into 4T1‐tumor‐bearing mice. C) The changes of temperature in the tumor area with the extension time of 660 nm NIR laser irradiation and thermal images at different times after TS NPs (up) or PBS (down) were injected into 4T1‐tumor‐bearing mice.

Inspired by the excellent phototherapeutic effects of TS NPs in vitro, we further evaluated the therapeutic effect in vivo. In the preliminary step, TS NPs were intravenously injected into the tumor‐bearing mice through the tail and monitored the NIR‐II fluorescence imaging in real‐time. As illustrated in **Figure** **3**A, the fluorescence signals at the tumor site showed a trend of enhancement after 3 h post‐injection of TS NPs, and the fluorescence intensity at the tumor site gradually increased over time, indicating that TS NPs could effectively accumulate at the tumor site by EPR effect. The fluorescence signal in the tumor area reached its plateau after 24 h injection, the signal‐to‐noise ratio (SNR) was determined to be 8.90 (Figure S22, Supporting Information), and then the signal gradually decreased over time (Figure S23, Supporting Information). After 48 h, the mice were dissected, major organs and tumors were isolated, and the biological distribution of TS NPs was further evaluated by NIR‐II FLI. It was found that the fluorescence signal in the tumor tissue area was still strong (Figure 3A), indicating that TS NPs can achieve long‐term retention in the area of the tumor and have great potential application in the surgical navigation guided by NIR‐II FLI. It should be noted that the fluorescence signals in the area of the liver could be resulted from the gradual metabolism of TS NPs after 48 h. However, tumor areas in PBS‐treated mice showed a negligible background fluorescence in the NIL‐II window (Figure S24, Supporting Information). At the same time, the photoacoustic signals of TS NPs in the tumor were observed. As shown in Figure 3B, the photoacoustic signals reached the strongest intensity after 24 h injection (SNR: 11.66), and then gradually weakened with the metabolism (Figure S22, Supporting Information). In addition, to evaluate the short‐term toxicity to liver of TS NPs, we collected serum of mice for hepatic function markers (alanine aminotransferase (ALT), aspartate aminotransferase (AST), and albumin (ALB)) analysis after 12 h, 24 h and 48 h after administration, respectively. The results demonstrated that the ALT, AST, and ALB markers in mice administrated with TS NPs showed no significant difference compared to the control mice administrated with PBS, suggesting that TS NPs had no short‐term toxicity to the liver (Figure S25, Supporting Information).

The in vivo photothermal effects of TS NPs were then evaluated using tumor‐bearing mice. At 24 h post‐injection, the tumor area of mice was irradiated by 660 nm (500 mW cm^−2^) laser for 10 min, and the temperature of the tumor area was monitored. As shown in Figure 3C, the temperature of the tumor area rapidly increased from 33 to 56.5 °C, and then reached a maximum in 3 min, which manifested the excellent photothermal performance of TS NPs in vivo. In contrast, in the control group (PBS+Light), the temperature in the area of the tumor raised only 5 °C, showed that the laser itself did not significantly change the temperature of the tumor. Subsequently, we further evaluated the effects of 660 nm laser on tumor volume and body weight in tumor‐bearing mice. Using PBS (dark treatment) as a control, it was found that there was nearly no difference in tumor volume and body weight in mice after laser irradiation (once time, 660 nm, 500 mW cm^−2^, 5 min) (Figure S26, Supporting Information). This further demonstrated that the laser only had a negligible effect on tumors.

**Figure 4:**
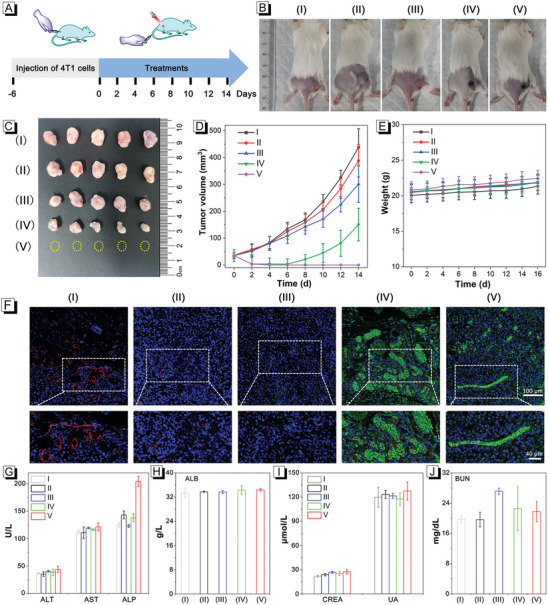
A) The schematic diagram of tumor‐bearing mice and the treatment process. B) Tumor images of mice after 14 days’ treatment. C) Anatomical images of tumors dissected from the sacrificed mice after treatment. Growth curve of tumor volume D) and body weight E) change with time in different treatment groups (n = 5). F) Histopathological view of the tumor tissue dissected from the mice after CD31, Dextran, and DAPI staining. G–J) Blood biochemistry indexes of ALT, AST, ALP, CREA, BUN, and ALB. Data are presented as the mean ± SD (n = 5).

The phototherapeutic effects in vivo of TS NPs were then conducted on the 4T1‐tumor‐bearing mice. First, 4T1 cells were injected into mice to construct model mice (at the −6th day). After 6 days (at the 0 day), when the volume of the subcutaneous tumor reached ≈ 40 mm^3^, different drugs were injected through tail vein, and the laser irradiation (once time, 660 nm, 500 mW cm^−2^, 5 min) was performed after 24 h (at the 1st day) (**Figure** **4**A). The mice with similar tumor size were divided into 5 groups: (I) PBS + L, (II) Sorafenib NPs, (III) TS NPs + D, (IV) DBD‐TM NPs + L (V) TS NPs + L. The weight of mice and the volume of tumors were measured every other day. It was observed that the volumes of tumor in group I–III gradually increased (Figure 4D). Although the volume of tumors in group IV was suppressed, it gradually increased in the later stage, and tumor recurrence occurred. In sharp contrast, for group V, the tumor did not recur in the treatment, solidly demonstrating that the TS NPs can achieve tumor elimination. Additionally, the weight of the mice remained essentially constant throughout the treatment (Figure 4E), and the results revealed the negligible systemic toxicity of these NPs. At the 14th day, the tumors in group V were obviously eliminated, while the tumor growth in the other groups (group I–III) was more obvious or recurrent (group IV) (Figure 4B,C).

Generally speaking, vascular collapse in organisms will lead to increased vascular permeability, decreased vascular perfusion and blood flow.^[^ ^14^ ^]^ Therefore, tumor vascular permeability was studied by measuring vascular leakage with FITC‐labeled dextran.^[^ ^15^ ^]^ The tumor areas of mice were all irradiated by laser for 5 min, after 24 h, FITC‐labeled dextran was injected intravenously, and tumors were collected 30 min later. The terminal deoxynucleotidyl transferase dUTP nick end labeling (TUNEL) and Ki67 assay exhibited a large number of apoptosis and minimal proliferation of tumor cells in the group V, revealing its effective killing ability (Figure S27, Supporting Information). Compared with PBS group, the leakage of dextran in tumor sections of treated mice (group IV and V) increased significantly, suggesting that the nanoparticles containing DBD‐TM could disrupt tumor blood vessels under laser irradiation, which was caused by the photothermal effect of DBD‐TM. Notably, the platelet endothelial cell adhesion molecule‐1 (CD31) assay revealed that the angiogenesis was significantly inhibited in the treatment group (group V) (Figure 4F). In addition, evident vascular inhibition was also observed in control group (group II and III), while relatively obvious angiogenesis was found in the group I and IV. Based on this phenomenon, it was further inferred that the treatment group (group V) achieved complete elimination of the tumor by inhibiting angiogenesis in the tumor area, killing tumor cells, and rupturing tumor blood vessels, speculating tumor recurrence in group IV was caused by neovascularization. That is to say, sorafenib, not only achieves synergistic therapeutic effects with DBD‐TM at tumor cells, but also has an inhibitory effect on angiogenesis in tumor tissues, further demonstrating the superiority of the combined therapy of sorafenib and DBD‐TM.

In addition, the in vivo biosafety of all treatment groups was further analyzed. Major organs of mice were taken for hematoxylin and eosin staining (H&E) staining. As displayed in Figure S28 (Supporting Information), no significant inflammation lesions or impairment was observed in H&E stained sections, further reflecting that toxicity in all experimental groups can be negligible. Finally, the blood was collected from all mice at the 14th day, and further analyzed the blood biochemical indices. As illustrated in Figure 4G–J, the contents of ALT, AST, alkaline phosphatase (ALP), ALB, creatinine (CREA), uric acid (UA), and blood urea nitrogen (BUN) were all remained within the normal range, demonstrating that the drugs used in this experimental scheme have good biocompatibility and low side effects.

## 3 Conclusion

In summary, we reported a versatile nanomaterial on the basis of both well‐tailored AIEgen and anti‐angiogenic agent, allowing multimodal imaging‐navigated synergistic therapy toward cancer. Through subtle regulation of structural conformation, D‐A strength, and intramolecular rotators, AIEgen DBD‐TM was constructed and exhibited remarkable NIR‐II fluorescence signals and prominent photothermal conversion behaviors upon single 660 nm laser irradiation, manifesting the distinctive balance between radiative and nonradiative excited‐state energy dissipations. By nanoprecipitation methods, TS NPs were fabricated by integrating DBD‐TM with the anti‐angiogenic agent sorafenib. TS NPs inherited the intrinsic features of DBD‐TM and sorafenib, and exhibited appropriate particle size as a nanodrug, good monodispersity and stability in aqueous phase, excellent biocompatibility, as well as good tumor‐targeting ability. In vitro and in vivo assessments revealed that TS NPs well performed in NIR‐II FLI‐PAI‐PTI trimodal imaging‐navigated PTT‐anti‐angiogenesis synergistic therapy, affording simultaneous accurate tumor diagnosis imaging and complete tumor elimination. Comparing to single phototheranostics protocol, the developed innovatory strategy involving anti‐angiogenesis is capable of affording superior tumor ablation and largely restrained tumor recurrence, thus representing an unprecedented theranostic formula beyond phototheranostics. It was firmly believed that these findings in this study would trigger state‐of‐the‐art developments of cancer theranostics in clinical trials.

## Conflict of Interest

The authors declare no conflict of interest.

## Supporting information

Supporting Information

## Data Availability

The data that support the findings of this study are available from the corresponding author upon reasonable request.
