# Supplementary material for: Regulating Aggregation‐Induced Emission Luminogen for Multimodal Imaging‐Navigated Synergistic Therapy Involving Anti‐Angiogenesis
Source: Adv Sci (Weinh). 2024 Aug 29;11(40):2302713. doi: 10.1002/advs.202302713 (PMC11515900; doi:10.1002/advs.202302713)
Supplement: Supplementary file 1 — Supporting Information [file ADVS-11-2302713-s001.docx]

Supporting Information

**Regulating Aggregation-Induced Emission Luminogen for Multimodal Imaging-****Navigated Synergistic Therapy Involving Anti-angiogenesis**

Fei Zhang, Jie Cui, Yao Zhang, Miao Yan, Xiaoxiao Wu, Xue Liu, Dingyuan Yan, Zhijun Zhang, Ting Han, Hui Tan,* Dong Wang,* and Ben Zhong Tang*

**Experimental Section**

**Main Materials:** Commercially available chemicals, such as 6-bromoindoline-2,3-dione, 6-bromoindolin-2-one, 9-(bromomethyl)nonadecane, phenylboronic acid, (4-(diphenylamino)phenyl)boronic acid, (4-(bis(4-methoxyphenyl)amino)phenyl)boronic acid, Pd(dppf)Cl_2_ were obtained from Adamas, Energy, TCI and Bide and used as received unless otherwise stated. Phosphate buffered saline (PBS), fetal bovine serum (FBS), penicillin/streptomycin, Hoechst 33342, LysoTracker Deep Red were purchased from Thermo Fisher Scientific. Roswell Park Memorial Institute (RPMI-1640) medium, Dulbecco's Modified Eagle's Medium (DMEM) medium, fetal bovine serum (FBS) were purchased from Gibco. All other chemicals were of analytical grade and used as received without further purification. Ultrapure water was supplied by Mill-Q Plus System (Millipore Corporation, USA).

**Instruments:** Reactions were monitored with analytical thin-layer chromatography (TLC) on silica. ^1^H NMR and ^13^C NMR data were recorded on Bruker nuclear resonance (500MHz) spectrometers unless otherwise specified, respectively. Chemical shifts (δ) are given in ppm relative to TMS. The MALDI-TOF-MS mass analysis was performed on an AXIMA-CFRTM plus instrument. UV-vis spectra and photoluminescence (PL) spectra were measured at room temperature in Shimadzu UV-2600 UV/Vis spectrophotometer and an Edinburgh FS1000 fluorescence spectrometer, respectively. Particle size analyses were implemented using a Zetasizer Nano ZSP. Photothermal experiments were implemented by using a 660 nm infrared semiconductor laser (Changchun radium photoelectric technology). Temperature changes were recorded by an E6 IR thermal camera (FLIR Systems). The fluorescent images of cell were taken by a confocal laser scanning microscope (CLSM, ZEISS-LSM900). All NIR-II images were collected on MARS in vivo imaging system (Artemis Intelligent Imaging, Shanghai, China). Live animal photoacoustic imaging were collected on Vevo LAZR (USA).

**Preparation of NPs:** 3 mL of THF solution containing 1 mg of compound DBD-TM, 1 mg of sorafenib, and 10 mg of DSPE-PEG_2000_ (m(DBD-TM) : m(sorafenib) : m(DSPE-PEG_2000_) = 1:1:10) was poured into 20 mL of deionized water. The ultrasound was then carried out continuously for 2 minutes at 45% output power with a micro-tip probe ultrasonic device. Then the mixtures were transferred into dialysis tube (MWCO 3500 Da) and dialyzed against deionized water for 24 h. In order to remove THF completely, the water was replaced by fresh water every 4 h. The final obtained nanoparticle solutions were concentrated by ultrafiltration.

**Photothermal performance measurement:** The aqueous solution of TS NPs was continuously exposed to a 660 nm laser at appointed power density. The temperature was measured every 5 s and stopped until the temperature nearly reached to a plateau. The corresponding infrared thermal images of the sample tubes were also acquired. In addition, pure water under the same condition served as the control groups.

**Cell lines and animal models:** The 4T1 cells were cultured in RPMI 1640 Medium (Gibco, NY, USA) containing 10% FBS, penicillin (50 U/mL) and streptomycin (50 mg/mL), at 37 °C in a 5% CO_2_ incubator. Female BALB/c mice (5-6 weeks old) were purchased from Guangdong Medical Laboratory Animal Center. All animal procedures strictly complied with the regulations of the Animal Ethical and Welfare Committee of Shenzhen University (AEWC-SZU) and conduct in Peking University Laboratory Animal Center of Shenzhen Graduate School. 1 × 10^6^ 4T1 cells were injected into the back of mice for tumor modeling. The tumor volumes of mice were measured every two days.

**Cellular internalization:** To track internalization of TS NPs in tumor cells, 4T1 cells (3 × 10^4^) were grown in glass-bottom cell culture dishes for 18 h, and then incubated with FITC@DBD-TM NPs for another 12 h. The cells were washed with PBS for three times, followed by costaining with Lyso-Tracker Deep Red and Hoechst 33342. After washing the samples with PBS, the fluorescence images were captured by f lambda mode of CLSM.

**Live-dead cell staining:** To evaluate the PTT efficiency of TS NPs on cells, 4T1 cells (8 × 10^3^) were grown on slices in a 96-well culture plate for 24 h and incubated with TS NPs for another 24 h, followed by an 660 nm laser (0.5 W cm^-2^ for 5 min) irradiation. Two hours after the treatment, the cells were costained with calcein-AM (30 min) and propidium iodide (15 min) for fluorescence images to verify the cell death with fluorescence microscope.

**Cytotoxicity and phototoxicity assay:** 4T1 cells (8 × 10^3^) were seeded in a 96-well culture plate for 12 h. Then the culture medium was replaced with fresh medium containing different concentrations of compounds for 12 h with or without laser irradiation at 660 nm (0.5 W cm^-2^ for 5 min). After further incubation for 12 h, the cells were washed with PBS for three times, followed by incubation with fresh serum-free medium containing 10 % MTT for 4 h in darkness. Then the absorbance value was measured at 450 nm with a microplate reader to detect cell viability relative to the control cells after different treatments.

***In vivo* NIR-II fluorescence imaging:** The 4T1 tumor-bearing mice were anesthetized and intravenously injected with TS NPs. In vivo NIR-II fluorescence imaging (through the long pass (LP) filter of 1000 nm) and the picture were captured at different time.

**Computational details:** All compounds at ground states were fully optimized with the density functional theory (DFT) method by Gaussian 09 program and B3LYP/6-31G (d) basis set.

**Signal-to-noise ratio (SNR) calculation:** For the SNR calculation, The images obtained at 24 h after injection, the optimal time node for TS NPs enrichment, were selected for calculation. For NIR-II fluorescence image at 24 h after TS NPs injection, the mean fluorescent signal of tumor region and the ear region (as background) were measured. For PA imaging, the PA signal of the image at 0 h post injection (as background) and the PA signal the image at 24 h after injection (as the signal) were measured, respectively.

**Method of administration and the dosage**

All the drugs injected into the mice were administered intravenously. The mass concentrations of all groups was 1mg/mL and the dosage was 100 μL. For TS NPs, due to the encapsulation rates of DBD-TM and sorafenib were almost the same, the final concentration is calculated as the sum of the mass concentrations of sorafenib (about 0.5 mg/mL) and DBD-TM (about 0.5 mg/mL) and the dose volume was 100 μL.

***In vivo* vascular leakage and perfusion assay**

The experiment for tumors CD31 and Dextran analyses was carried out by referring to the previous study.^[1]^ When the tumor volume reached to 50 mm^3^, the mice were treated with (I) PBS, (II) Sorafenib NPs, (III) TS NPs, (IV) DBD-TM NPs, (V) TS NPs (at the 0 day). After 24 h (at the first day), group I, IV, and V are treated with light irradiation (660 nm, 500 mW cm^−2^, 5 min), groups II, and III do nothing. After another 24 h (at the second day), the vascular leakage was studied by intravenous injection of FITC-dextran (70 kDa, 25 mg mL^−1^, 100 μL). Thirty minutes after the injection, mice were sacrificed and tumors were collected. The tumor sections were stained with anti-CD31 antibody for the blood vessels (red) and DAPI for the cell nucleus (blue).

**Statistical analysis**

The experimental data were presented as means value ± standard deviation (SD). Image processing and mean intensity statistics were performed using Image J.

**Synthesis and characterization**

**Synthetic route of compound 1:** 75 mL of AcOH and 0.5 mL of concentrated hydrochloric acid was to a 250 mL three-way round-bottomed flask. Then 6-Bromoindoline-2,3-dione (2.26 g, 10.00 mmol) and 6-bromoindolin-2-one (2.12 g, 10.00 mmol) were added. Reflux for 24 hours, precipitate red solid. The mixture is cooled and filtered to get solid, and then the solid is washed three times with ethanol and ethyl acetate, and dried under vacuum to get pure product with a yield of 95%.

**Synthetic route of compound 2:** Compound 1 (2.1 g, 5.00 mmol), potassium carbonate (3.45 g, 25.00 mmol), DMF (100 mL) were added to a 250 mL three-necked round-bottom flask. Then 9-(bromomethyl)nonadecane (4.34g, 12.00 mmol) was added under the nitrogen atmosphere. The mixture was stirred at 100 ºC for 15 h until the reaction was complete. The mixture was then dissolved in CH_2_Cl_2_ and extracted three times with a large amount of saturated salt water. The excess DMF was removed, the organic phase was collected, dried with anhydrous Na_2_SO_4_ and drained under vacuum. Column chromatography (V_dichloromethane_: V_petroleum ether_ =1:10) to obtain the pure product, the product is oil liquid, yield 82%. ^1^H NMR (600 MHz, CDCl_3_): 9.07 (s, 1H), 9.05 (s, 1H), 7.16 (d, *J* =8.61 Hz, 2H), 6.89 (d, *J* =1.32 Hz, 2H), 3.62 (d, *J* =7.45 Hz, 4H), 1.88 (d, *J* =5.40 Hz, 2H), 1.30 (m, 64H), 0.88 (m, 12H).

**Synthetic route of DBD-P:** Compounds 2 (0.981 g, 1.00 mmol), phenylboronic acid (0.268 g, 2.20 mmol), [1,1 '-Bis(diphenylphosphino) ferrocene]dichloropalladium (7.00 mg, 1 mol%), 10 mL DMF, 2 M K_2_CO_3_ (10 mL) were added in 100 mL three-necked round-bottom flask. Reflux for 12 h at 100 ºC under nitrogen atmosphere. Then, the solvent is drained under vacuum. The product was extracted three times with dichloromethane and distilled water, and the organic phase was dried and concentrated with anhydrous Na_2_SO_4_. Column chromatography (V _dichloromethane_: V _petroleum ether_ = 1:1) to obtain the target product DBD-P, blue solid (0.75g, yield: 78%). ^1^H NMR (500 MHz, CDCl_3_): 9.20 (s, 1H), 9.18 (s, 1H), 7.58 (d, *J* = 7.65 Hz, 4H), 7.41 (t, *J* = 7.68 Hz, 4H), 7.33 (t, *J* = 7.28 Hz, 2H), 7.22 (m, 2H), 6.93 (d, *J* = 1.36 Hz, 2H), 3.67 (d, *J* = 7.36 Hz, 4H), 1.91 (t, *J* = 5.32 Hz, 2H), 1.21 (m, 64H), 0.78 (m, 12H). ^13^C NMR (126 MHz, CDCl_3_): 168.71, 145.81, 145.04, 140.64, 132.64, 130.12, 128.94, 128.18, 127.03, 120.98, 120.89, 44.60, 36.33, 31.92, 31.89, 31.70, 30.04, 29.72, 29.66, 29.64, 29.61, 29.36, 29.31, 26.58, 22.69, 22.67, 14.11.

**Synthetic route of DBD-T:** The synthesis of DBD-T was similar with DBD-P, affording product as a blue solid powder (yield: 55%). ^1^H NMR (500 MHz, CDCl_3_): 9.22 (s, 1H), 9.20 (s, 1H), 7.53 (d, *J* = 8.70 Hz, 4H), 7.30 (d, *J* = 2.28 Hz, 2H), 7.29 (d, *J* = 1.08 Hz, 4H), 7.27 (t, *J* = 1.82 Hz, 2H), 7.26 (d, *J* = 1.68 Hz, 1H), 7.24 (d, *J* = 1.62 Hz, 1H), 7.17 (t, *J* = 3.12 Hz, 4H), 7.15 (t, *J* = 3.26 Hz, 6H), 7.10 (t, *J* = 7.32 Hz, 2H), 7.08 (t, *J* = 1.12 Hz, 1H), 7.06 (t, *J* = 1.38 Hz, 2H), 7.05 (t, *J* = 1.00 Hz, 1H), 6.97 (d, *J* = 1.46 Hz, 2H), 3.73 (d, *J* = 7.40 Hz, 4H), 1.97 (d, *J* = 5.42 Hz, 2H), 1.37 (m, 12H), 1.21 (m, 52H), 0.83 (m, 12H). ^13^C NMR (126 MHz, CDCl_3_): 168.86, 148.12, 147.42, 145.76, 144.24, 133.90, 132.14, 130.01, 129.39, 127.64, 124.83, 123.39, 123.16, 120.58, 120.11, 105.90, 44.58, 36.36, 31.91, 31.87, 31.70, 30.05, 29.72, 29.65, 29.62, 29.60, 29.35, 29.30, 26.59, 22.69, 22.66, 14.14, 14.12. MS (MALDI-TOF): C_92_H_117_N_4_O_2_^+^ calcd [M+H]^+^ m/z, 1309.917; found m/z, 1309.087.

**Synthetic route of DBD-TM:** The synthesis of DBD-TM was similar with DBD-T, affording product as a blue solid powder (yield: 46%). ^1^H NMR (500 MHz, CDCl_3_): 9.19 (s, 1H), 9.17 (s, 1H), 7.48 (d, *J* = 7.56 Hz, 4H), 7.24 (d, *J* = 8.38 Hz, 2H), 7.12 (d, *J* = 7.84 Hz, 8H), 6.99 (t, *J* = 7.42 Hz, 4H), 6.94 (s, 2H), 6.87 (d, *J* = 7.74 Hz, 8H), 3.81 (s, 12H), 3.70 (d, *J* = 7.58 Hz, 4H), 1.96 (d, *J* = 5.20 Hz, 2H), 1.36 (m, 12H), 1.21 (m, 52H), 0.83 (m, 12H). ^13^C NMR (126 MHz, CDCl_3_): 168.93, 156.22, 145.70, 144.36, 140.49, 131.91, 127.45, 126.96, 120.32, 120.02, 119.83, 114.80, 55.51, 36.36, 31.91, 31.87, 31.69, 30.05, 29.72, 29.65, 29.62, 29.60, 29.35, 29.30, 26.58, 22.70, 22.66, 14.14, 14.12. MS (MALDI-TOF): C_96_H_125_N_4_O_6_^+^ calcd [M+H]^+^ m/z, 1430.962; found m/z, 1429.575.

**Scheme S1**. The structures and synthetic routes of **DBD-P**, **DBD-T** and **DBD-TM**.


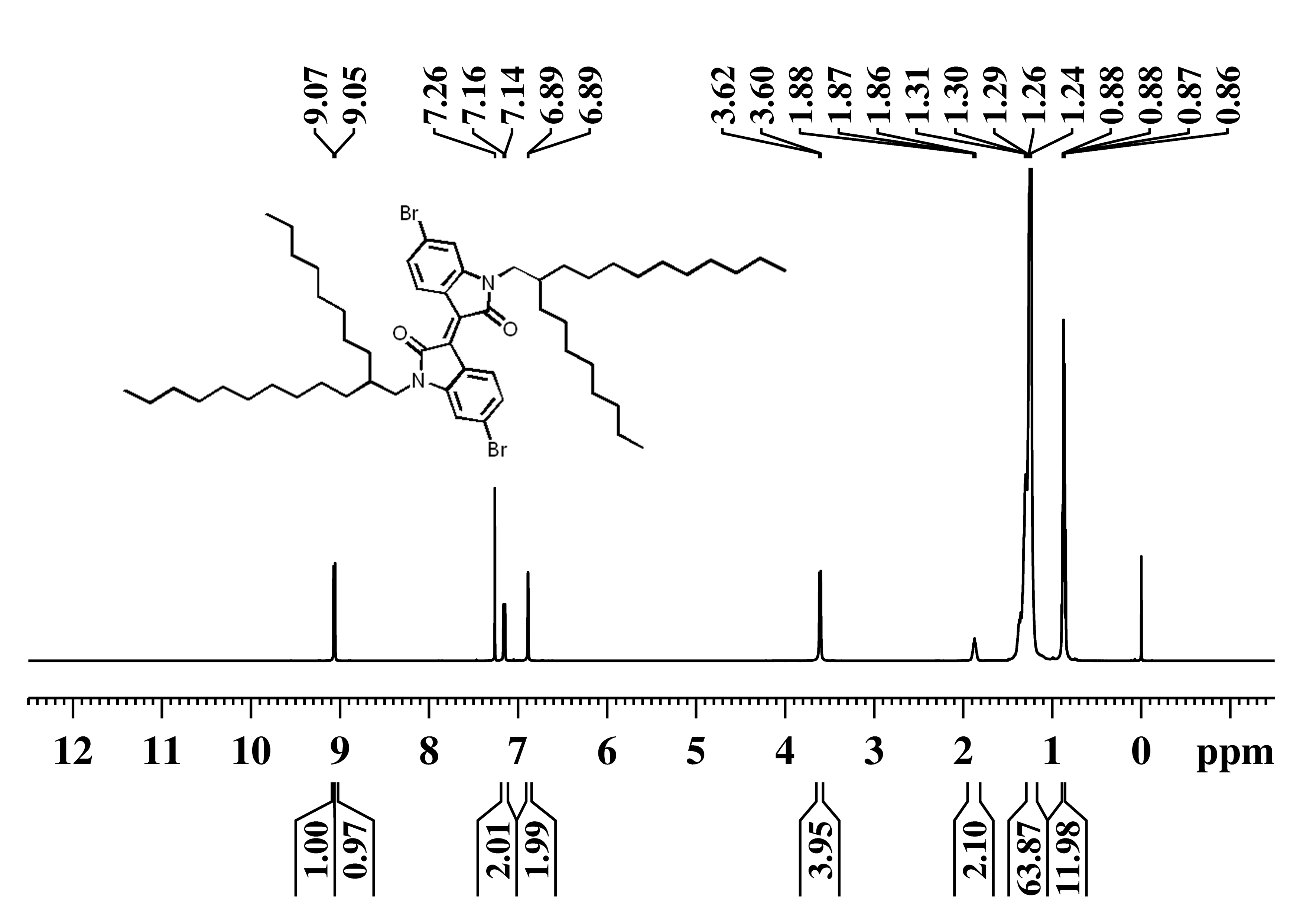


Figure S1. ^1^H NMR spectrum of (E)-6,6'-dibromo-1,1'-bis(2-octyldodecyl)-[3,3'-biindolinylidene]-2,2'-dione.


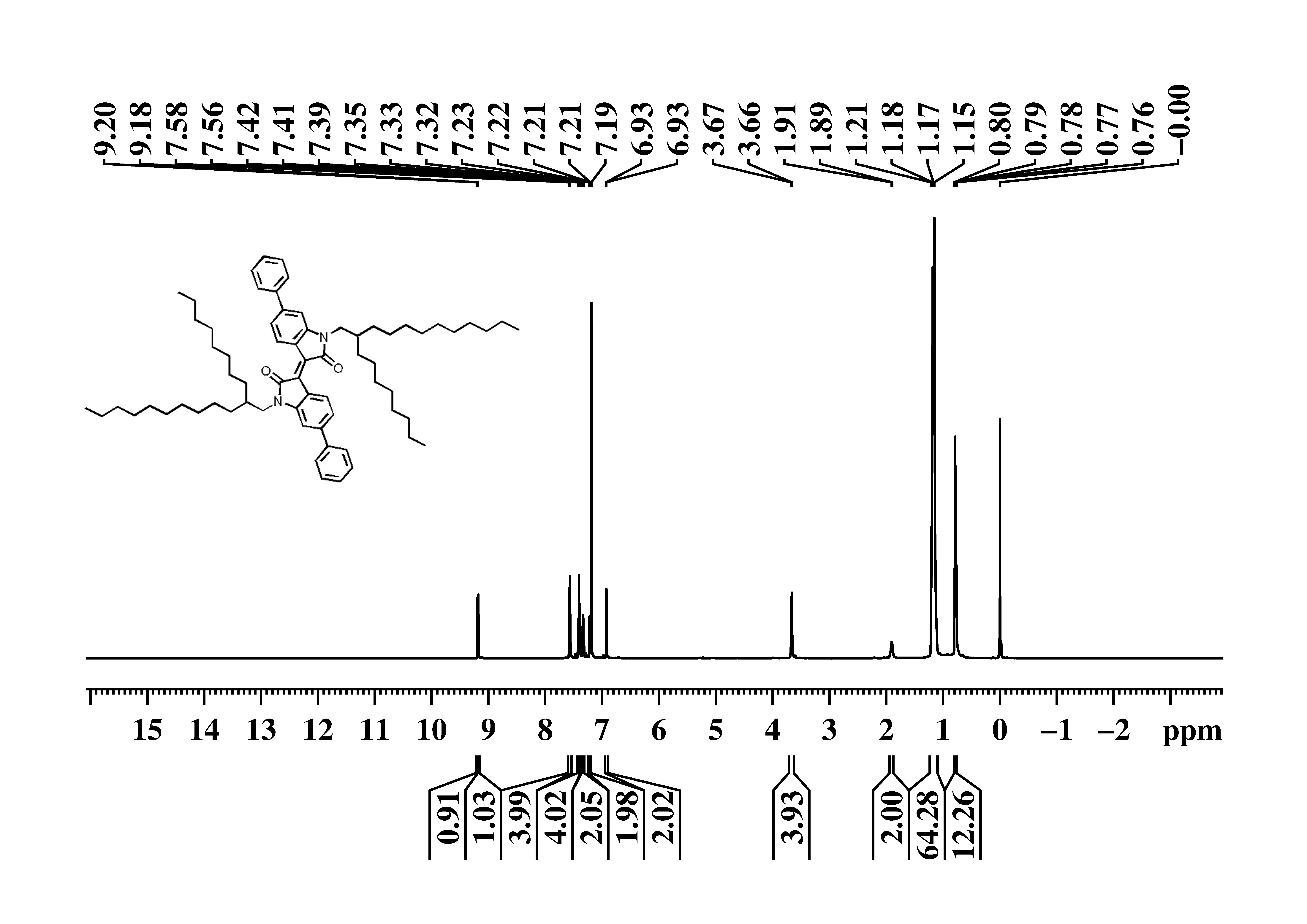


Figure S2. ^1^H NMR spectrum of (E)-1,1'-bis(2-octyldodecyl)-6,6'-diphenyl-[3,3'-biindolinylidene]-2,2'-dione.


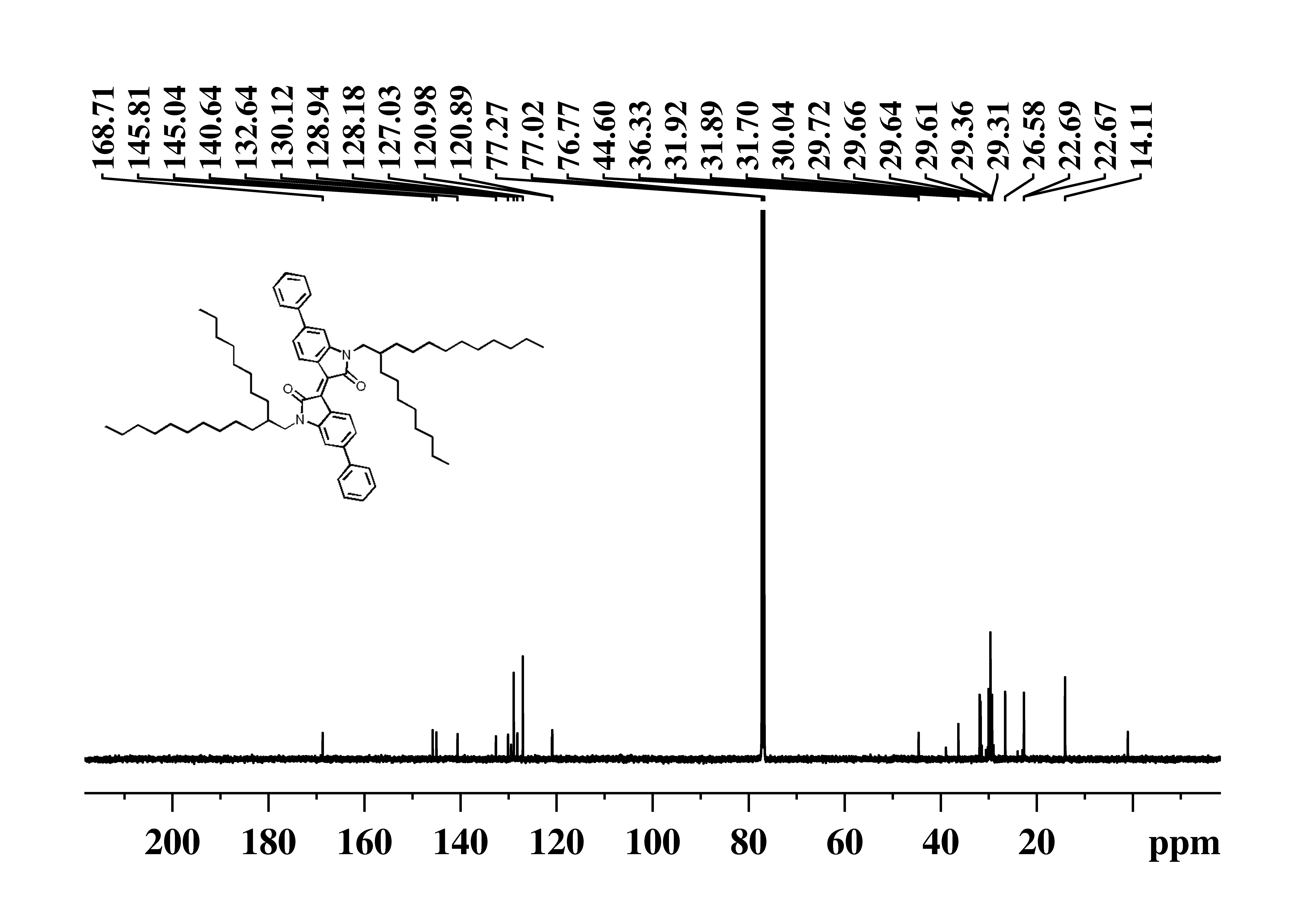


Figure S3. ^13^C NMR spectrum of (E)-1,1'-bis(2-octyldodecyl)-6,6'-diphenyl-[3,3'-biindolinylidene]-2,2'-dione.


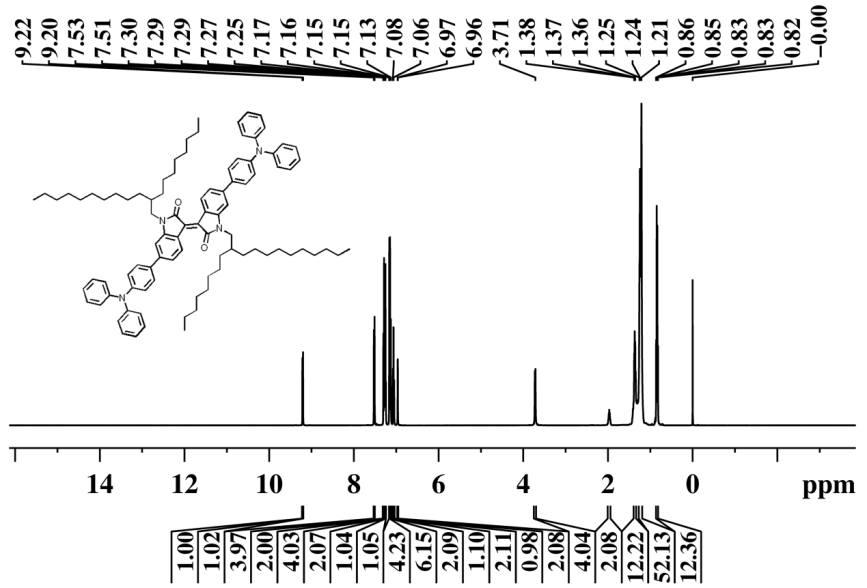


Figure S4. ^1^H NMR spectrum of (E)-6,6'-bis(4-(diphenylamino)phenyl)-1,1'-bis(2-octyldodecyl)-[3,3'-biindolinylidene]-2,2'-dione.


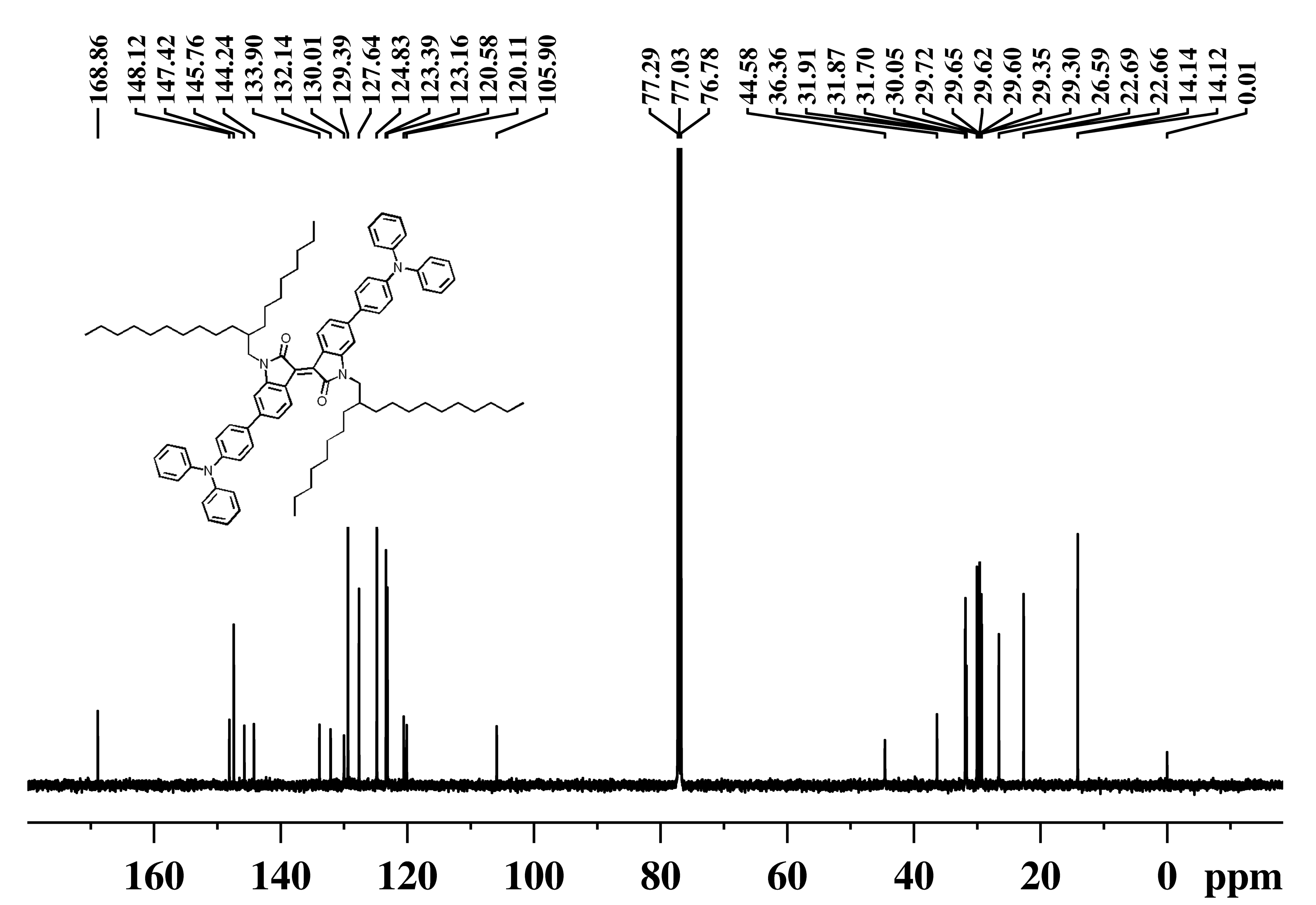


Figure S5. ^13^C NMR spectrum of (E)-6,6'-bis(4-(diphenylamino)phenyl)-1,1'-bis(2-octyldodecyl)-[3,3'-biindolinylidene]-2,2'-dione.

**
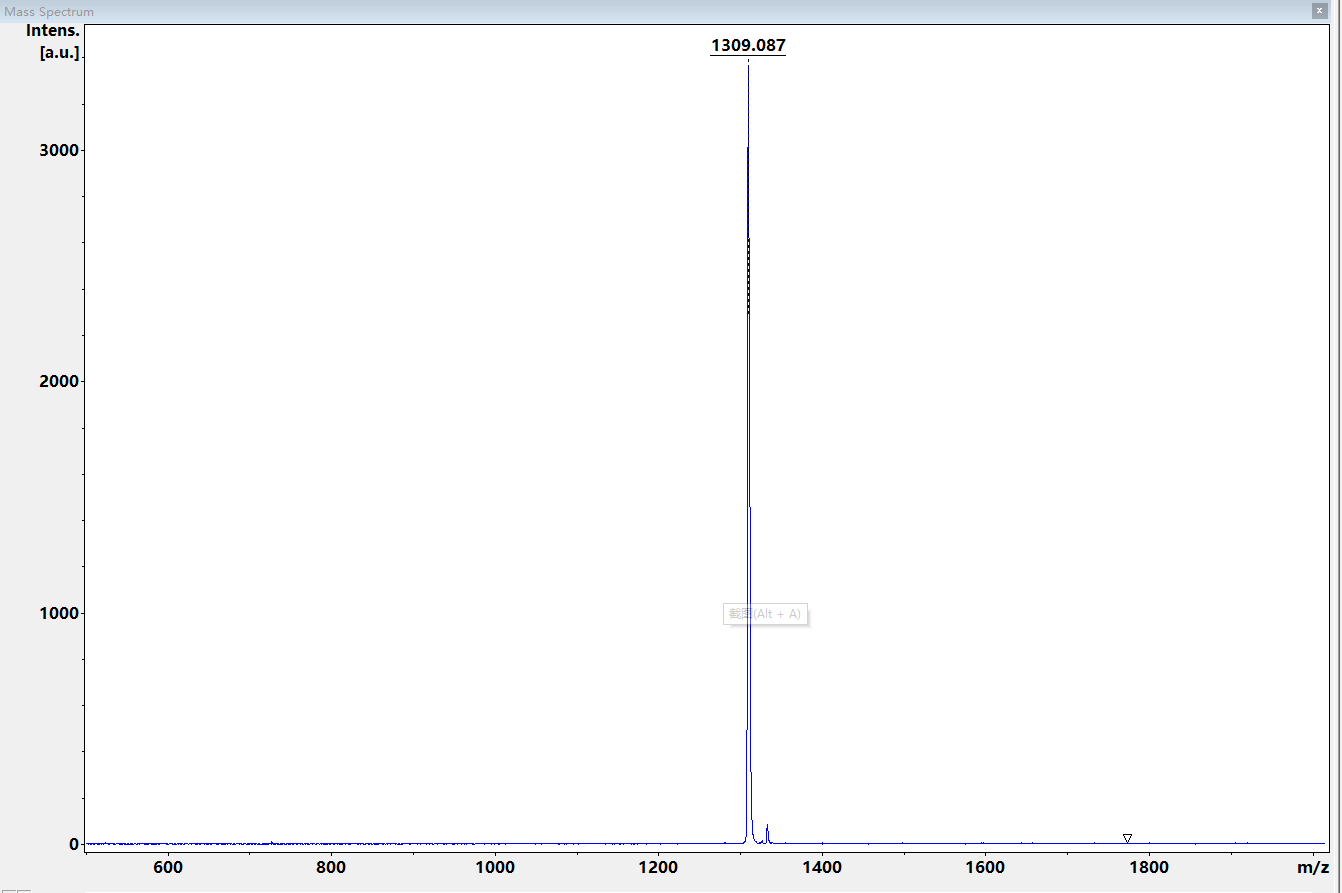
**

**Figure S6**. HRMS spectrum of (E)-6,6'-bis(4-(diphenylamino)phenyl)-1,1'-bis(2-octyldodecyl)-[3,3'-biindolinylidene]-2,2'-dione.


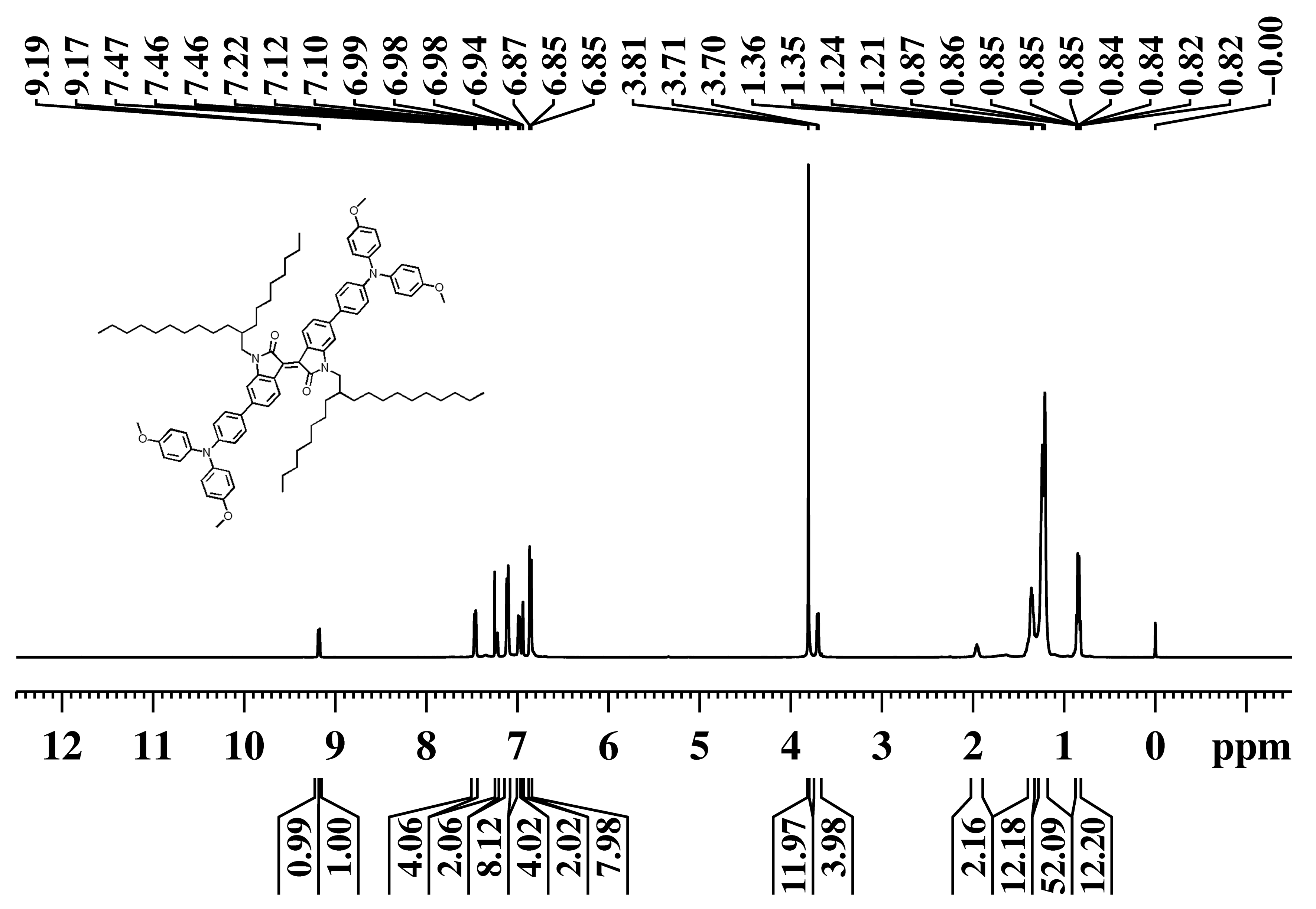


Figure S7. ^1^H NMR spectrum of (E)-6,6'-bis(4-(bis(4-methoxyphenyl)amino)phenyl)-1,1'-bis(2-octyldodecyl)-[3,3'-biindolinylidene]-2,2'-dione.


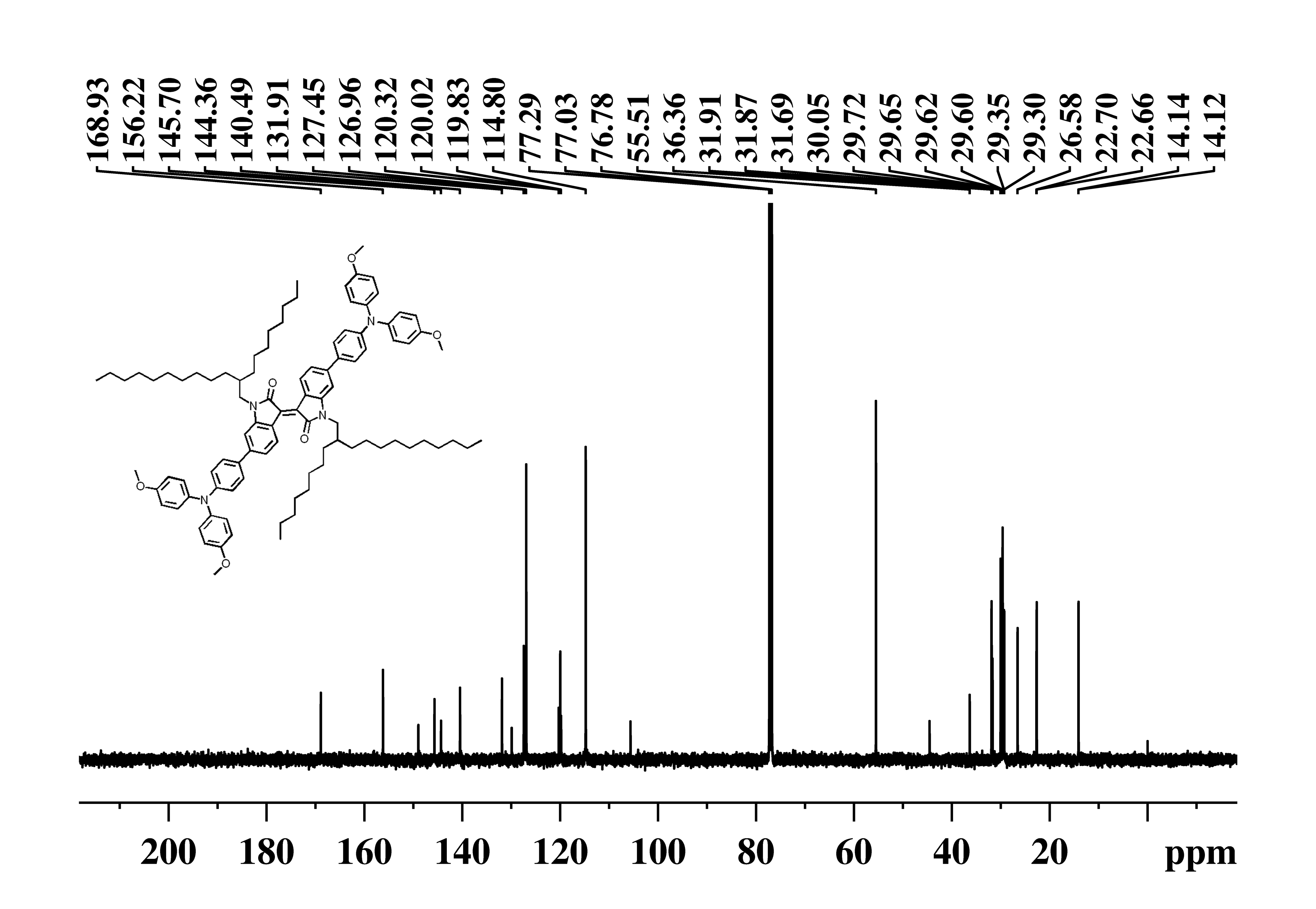


Figure S8. ^13^C NMR spectrum of (E)-6,6'-bis(4-(bis(4-methoxyphenyl)amino)phenyl)-1,1'-bis(2-octyldodecyl)-[3,3'-biindolinylidene]-2,2'-dione.

**
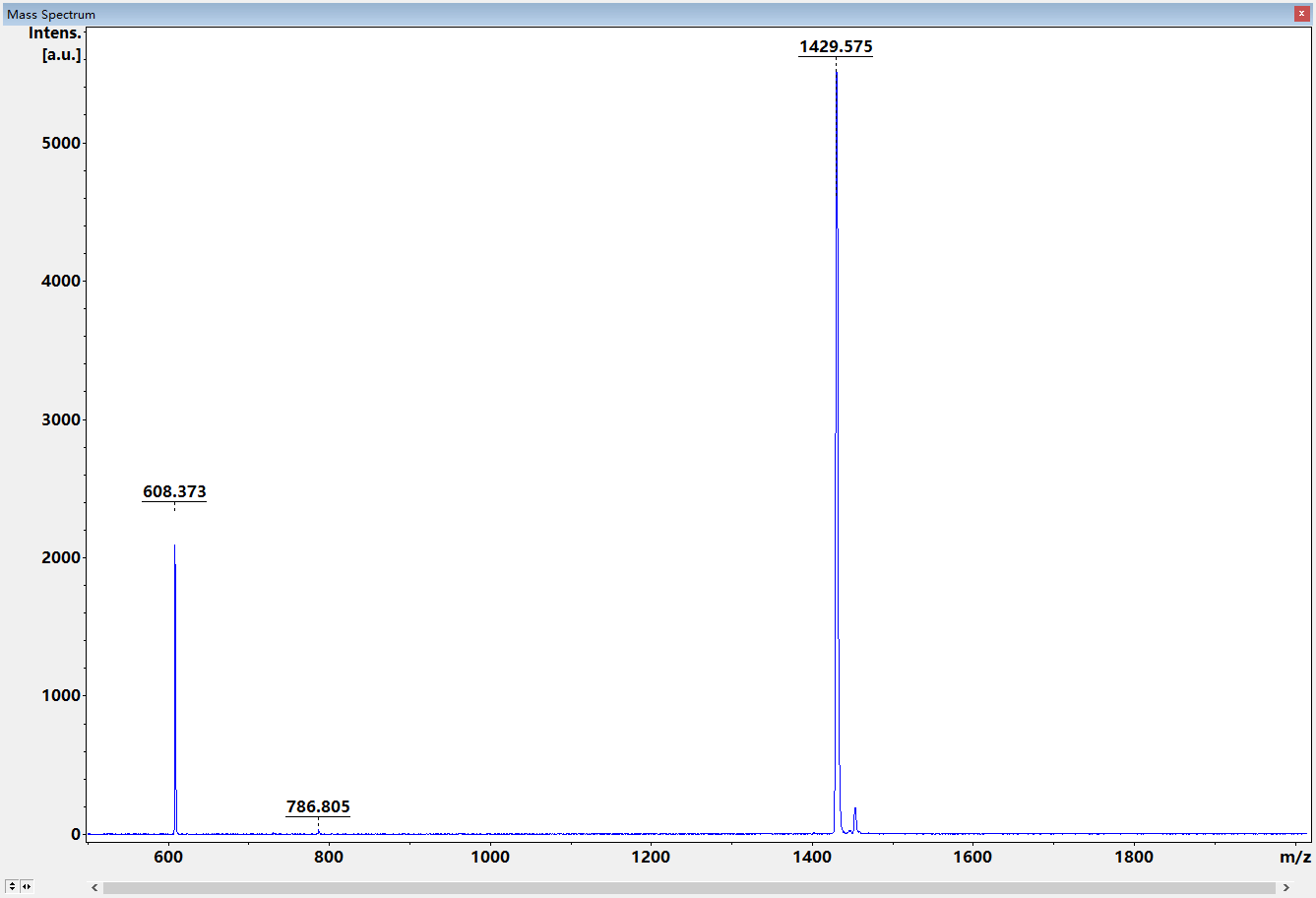
**

Figure S9. HRMS spectrum of (E)-6,6'-bis(4-(bis(4-methoxyphenyl)amino)phenyl)-1,1'-bis(2-octyldodecyl)-[3,3'-biindolinylidene]-2,2'-dione.


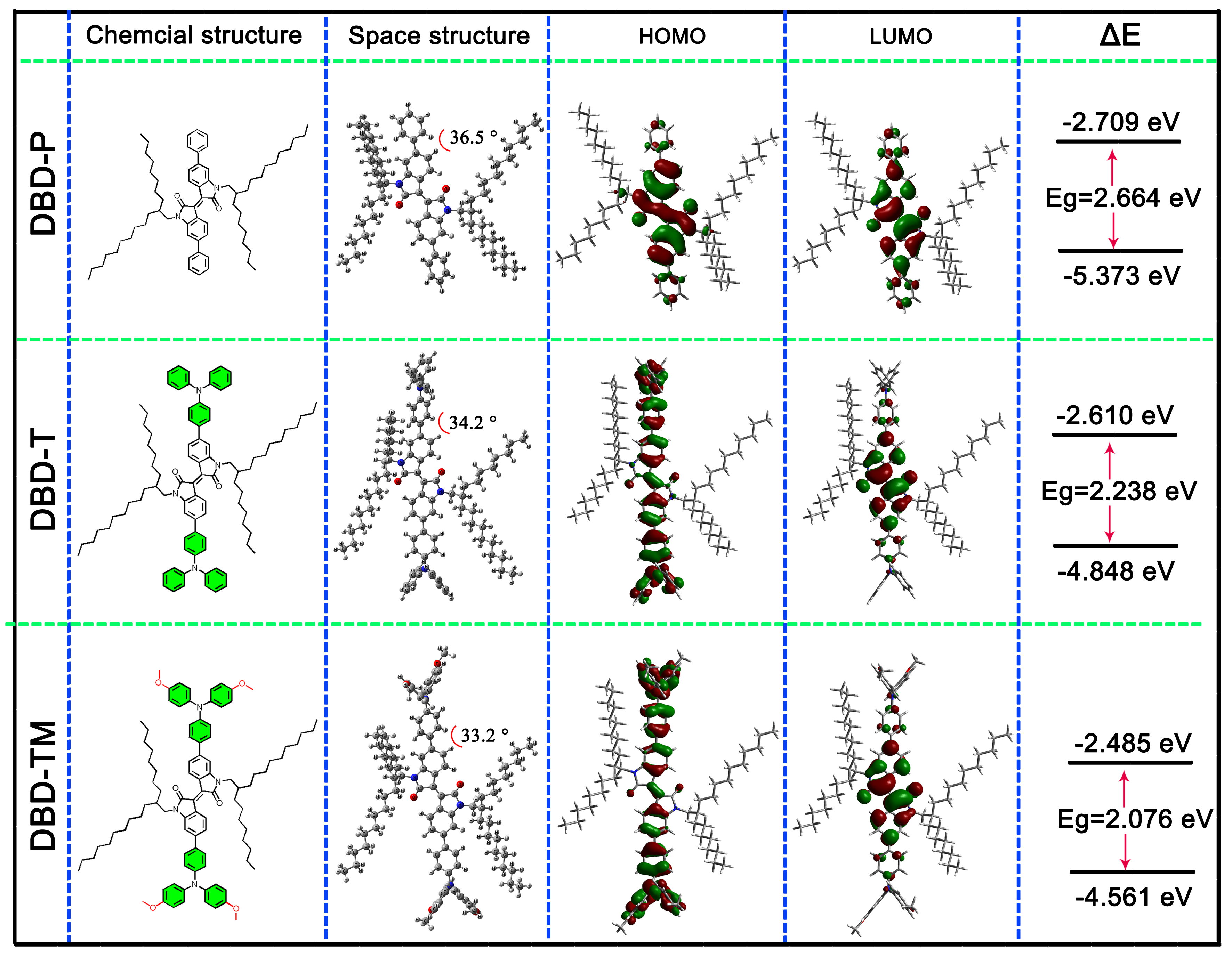


**Figure S10.** Chemical structures and molecular maps of **DBD-T** and **DBD-TM** determined at the B3LYP/6-31G(d) level.


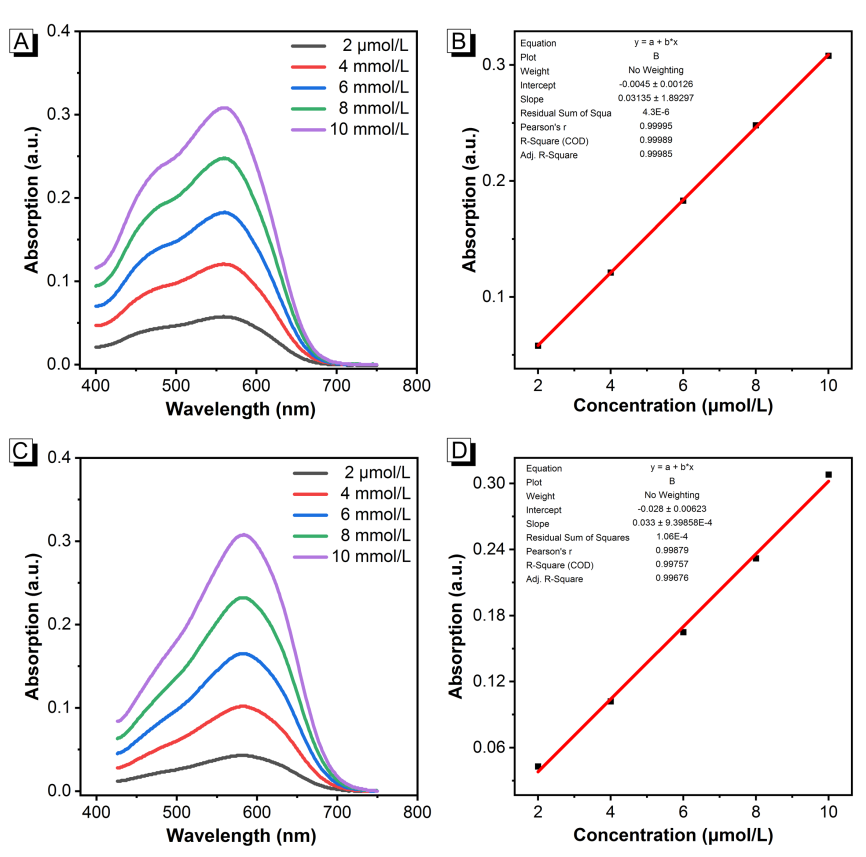


**Figure S11**. (A) Absorption curves of **DBD-T** in THF at different concentrations. (B) Linear absorbance versus concentration obtained from (A). (C) Absorption curves of **DBD-TM** in THF at different concentrations. (D) Linear absorbance versus concentration obtained from (C).


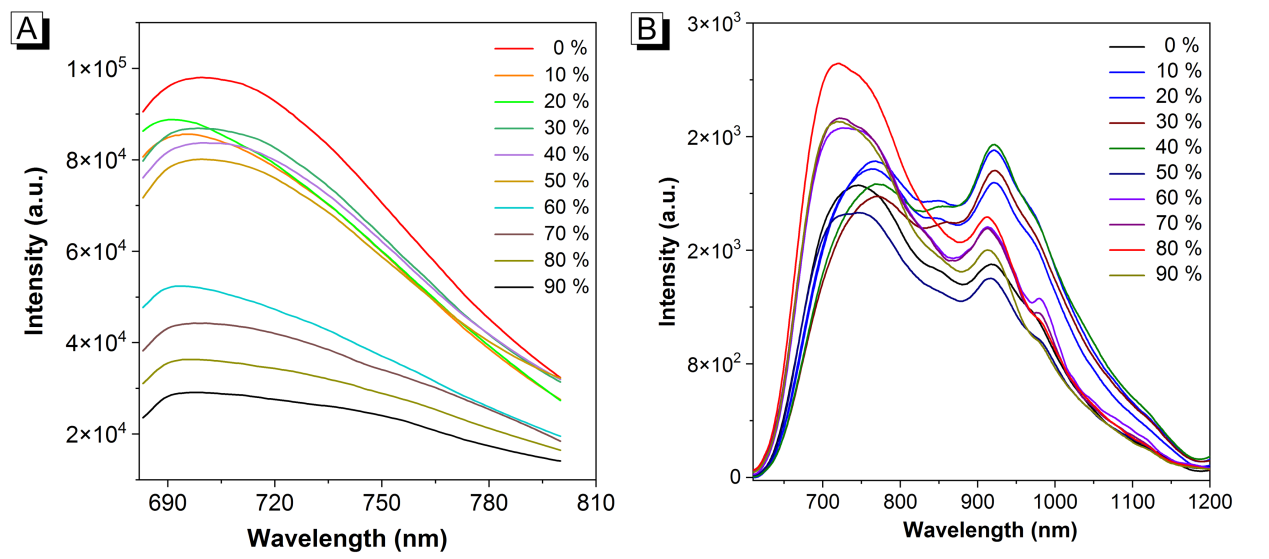


**Figure S12**. PL spectra of **DBD-P** (1.0 × 10^−6^ M, λ_ex_: 528 nm) (A) and **DBD-TM** (1.0 × 10^−6^ M, λ_ex_: 562 nm) (B) in tetrahydrofuran solution with different water fractions.


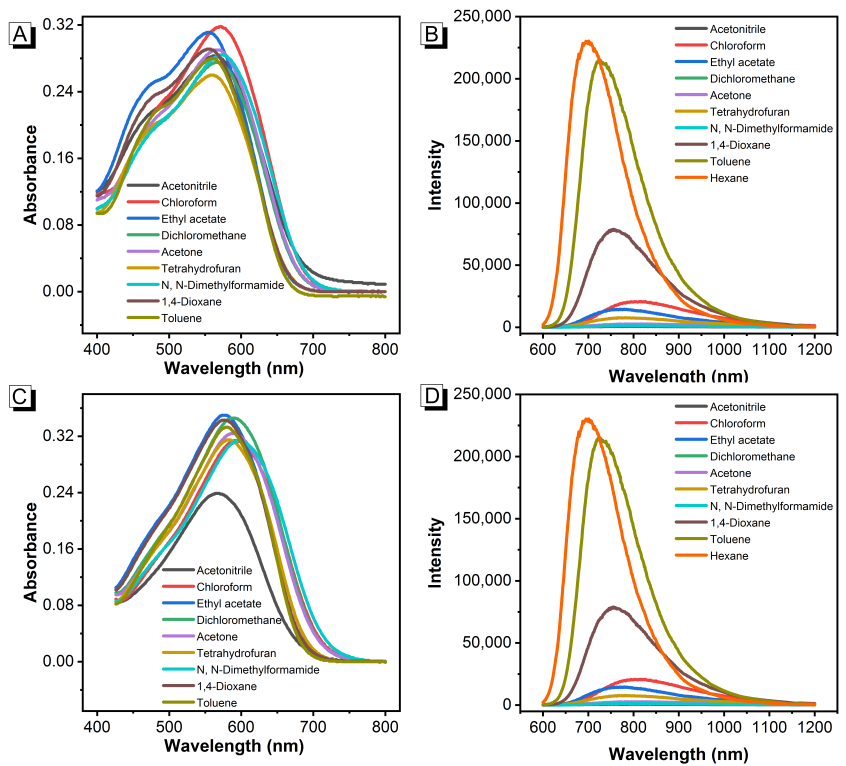


Figure S13. Absorption and emission spectra of (A, B) **DBD-T**, (C, D) **DBD-TM** in different solvents; Concentration: 10 μM.


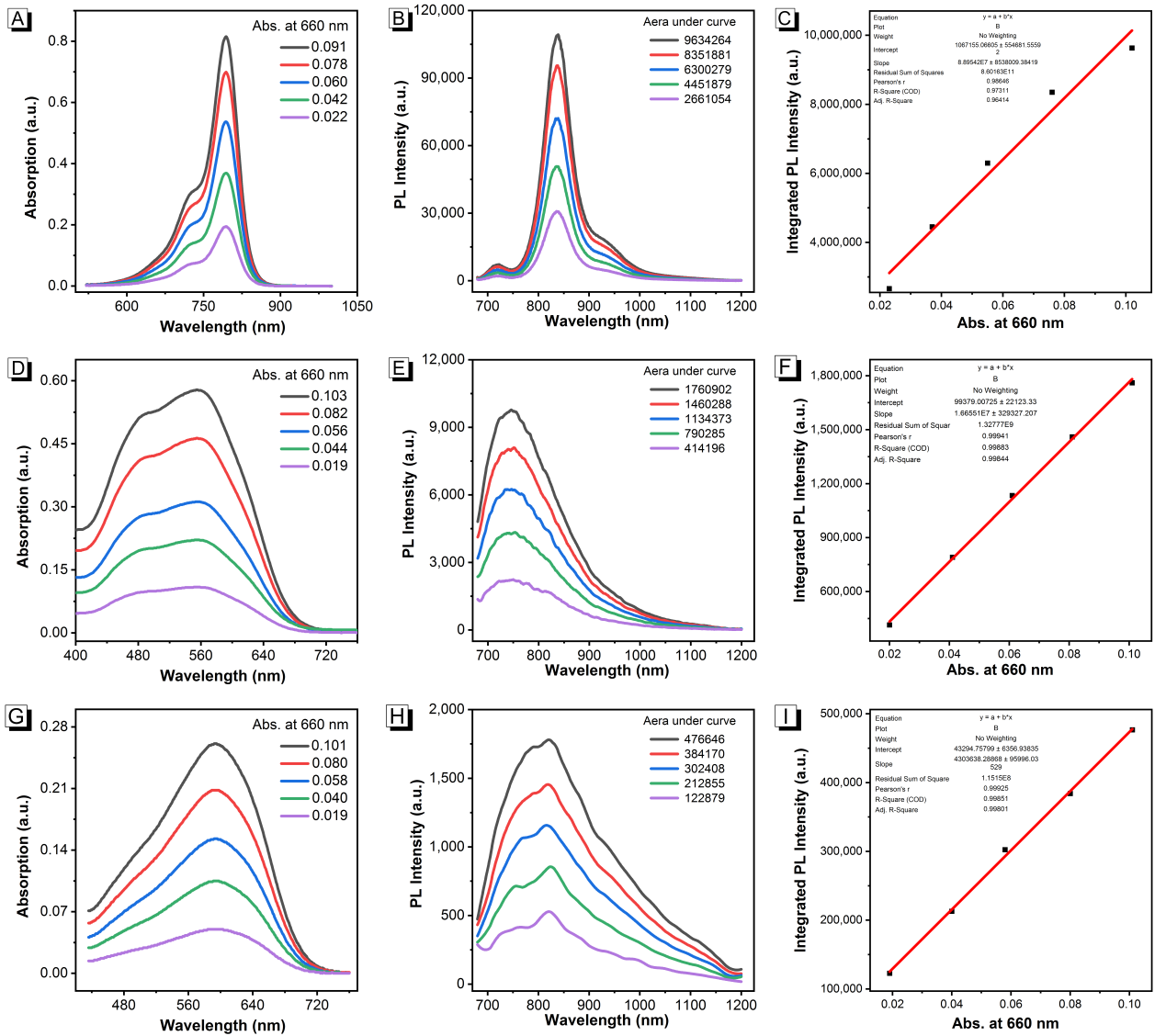


**Figure S14.** Fluorescence quantum yield measurement of **DBD-T** and **DBD-TM** in THF. UV-Vis-NIR absorption spectra (A, D, G) and PL spectra (B, E, H) at the excitation wavelength of 660 nm for ICG (A, B) in dimethyl sulfoxide, **DBD-T** (D, E), and **DBD-TM** (G, H) in THF. ICG, reference, QY = 13% in dimethyl sulfoxide.


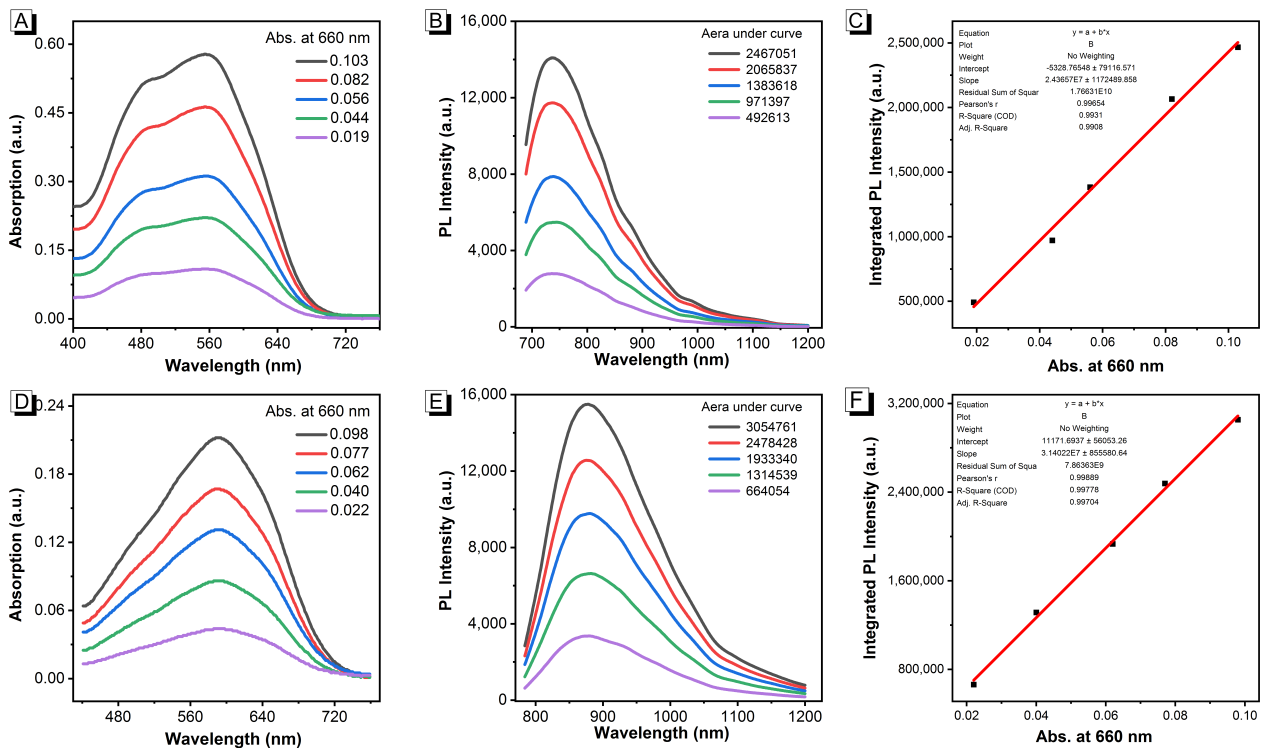


**Figure S15.** Fluorescence quantum yield measurement of nanoparticles (**DBD-T** NPs and **DBD-TM** NPs) in water. UV-Vis-NIR absorption spectra (A, D) and PL spectra (B, E) at the excitation wavelength of 660 nm for **DBD-T** NPs (A, B), and **DBD-TM** NPs (D, E) in water.


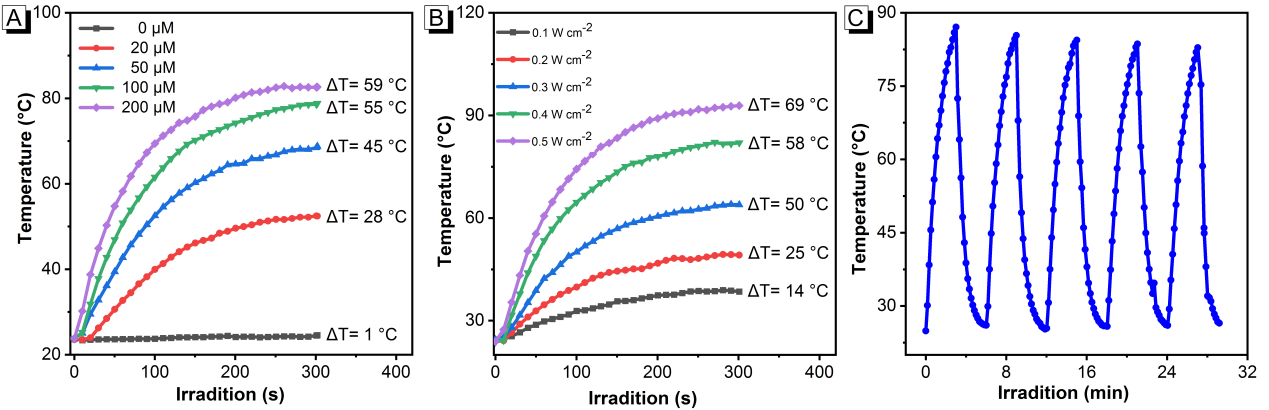


**Figure S16**. (A) Heating curves of **DBD-T** at different concentrations (0–200 μM) in dimethyl sulfoxide under 660 nm laser (0.3 W cm^-2^) illumination. (B) Corresponding heating curves of **DBD-T** in the concentrations of 100 μM in dimethyl sulfoxide under 660 nm laser irradiation with different exposure intensity (0.1–0.5 W cm^-2^). (C) Photothermal stability of **DBD-T** (100 μM) in dimethyl sulfoxide during five on/off irradiation cycles with a 660 nm laser at a power density of 0.5 W cm^-2^.


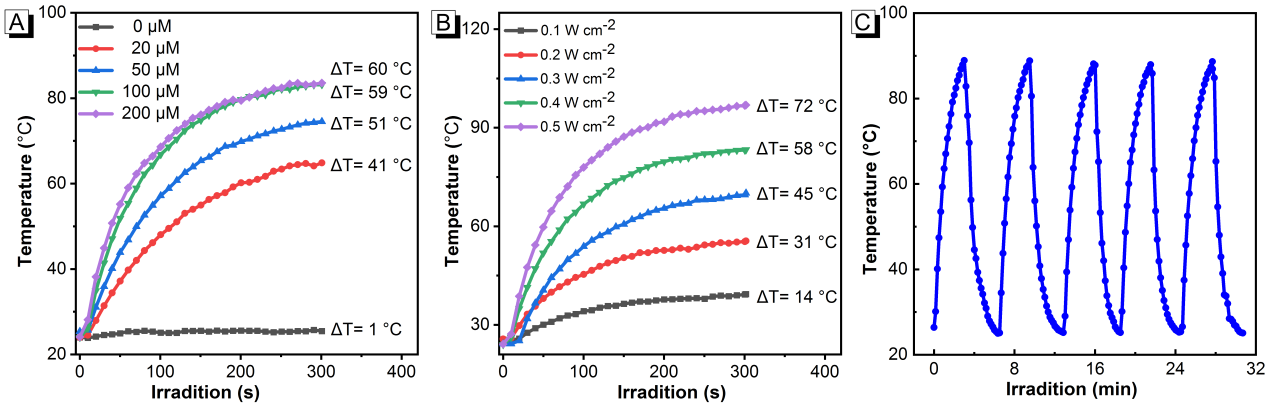


**Figure S17**. (A) Heating curves of **DBD-TM** at different concentrations (0–200 μM) in dimethyl sulfoxide under 660 nm laser (0.3 W cm^-2^) illumination. (B) Corresponding heating curves of **DBD-TM** in the concentrations of 100 μM in dimethyl sulfoxide under 660 nm laser irradiation with different exposure intensity (0.1–0.5 W cm^-2^). (C) Photothermal stability of **DBD-TM** (100 μM) in dimethyl sulfoxide during five on/off irradiation cycles with a 660 nm laser at a power density of 0.5 W cm^-2^.

**Table S1.** Some photophysical properties of **DBD-T** and **DBD-TM**. The absorption wavelength was measured in THF (1.0 × 10^−5^ M), the molar absorption coefficients were measured in THF, I/I_0_ was determined in 95% water proportion of THF. The photothermal conversion temperature is calculated by laser irradiation (660 nm, 0.3 W cm^-2^, 100 μM, 5 min).

| Compounds | | λ_abs_ | QYs (in THF) | QYs (nanoparticles) | ɛ/660 nm (10^4^ M^-1^ cm^-1^) | | AIE (*I/I_0_*) | Photothermal temperature (∆T) | |
| --- | --- | --- | --- | --- | --- | --- | --- | --- | --- |
| **DBD-T** | 572 nm | | 0.246% | 0.323% | 0.75 | 1.07 | | | 45 °C |
| **DBD-TM** | 598 nm | | 0.063% | 0.416% | 1.9 | 4.96 | | | 50 °C |


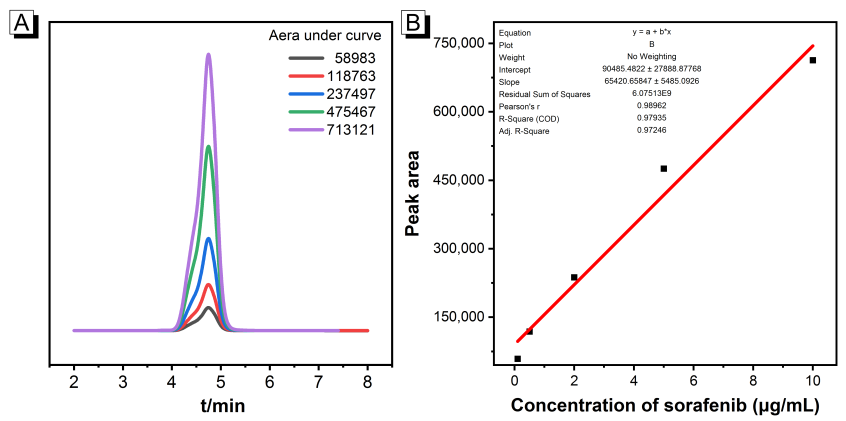


**Figure S18**. (A) HPLC-MS curves of sorafenib in methanol at different concentrations (0.2 μg/mL, 0.5 μg/mL, 2.0 μg/mL, 5.0 μg/mL, 10.0 μg/mL). (Mobile phase: acetonitrile/ammonium acetate (10 mmol/L) in solution (all containing 0.5% formic acid) = 80/20, flow rate: 0.25 mL/min.) (B) Linear aera of curves versus concentration obtained from (A).


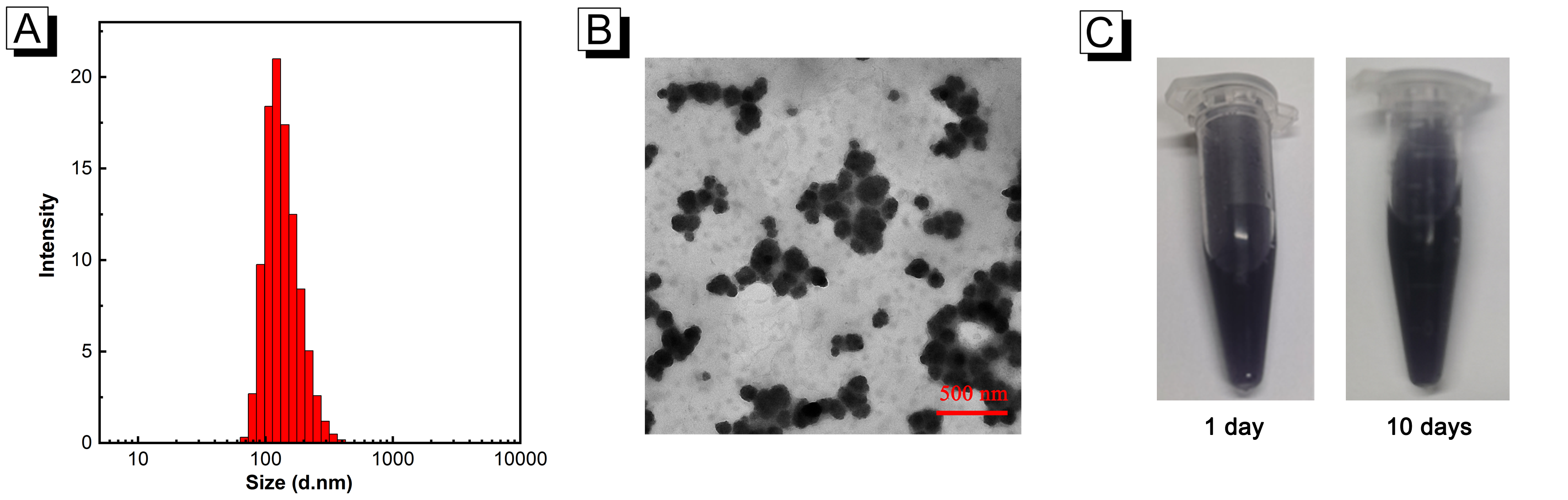


**Figure S19.** (A) DLS imaging of **TS** NPs in PBS, (B) TEM image of **TS** NPs, (C) photographs of **TS** NPs standing for 1 day and 10 days.


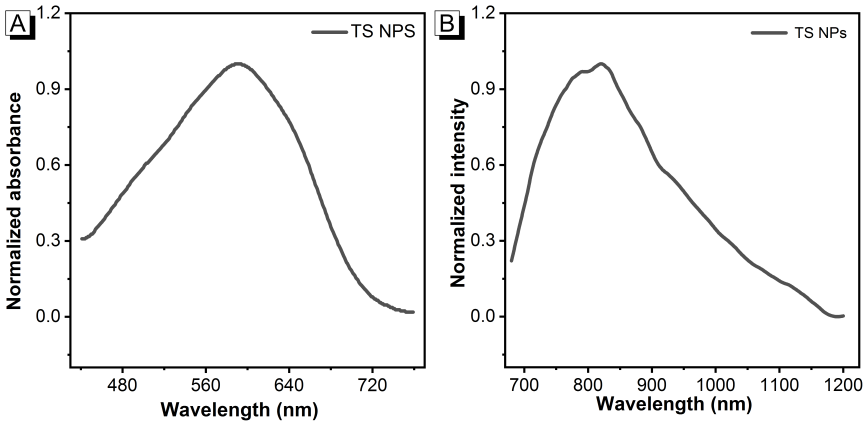


**Figure S20**. Normalized UV absorption spectra (A) and fluorescence emission spectra (B) of **TS** NPs.


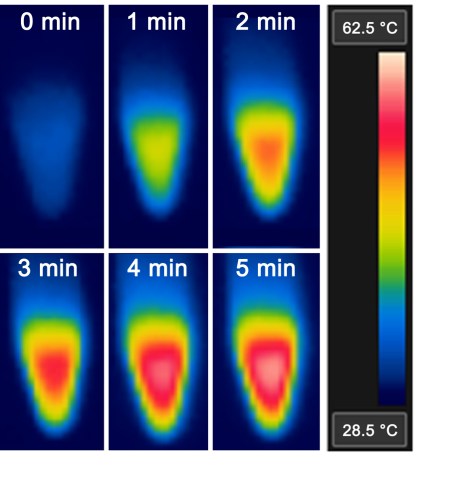


**Figure S21**. The infrared ray thermal images of TS NPs (100 μM, 300 mW cm^-2^) in PBS under different time irradiation.

Table S2. IC_50_ and Chou-Talalay combination index (CI) synergy analysis.

|  | IC_50_ values | | CI values |
| --- | --- | --- | --- |
|  | sorafenib NPs (μM) | **TM2** NPs (μM) |  |
| sorafenib NPs  **TM2** NPs +L  **TS** NPs +L | 37.68 | / | / |
|  | / | 45.08 | / |
|  | 18.18 | 3.61 | 0.56 |


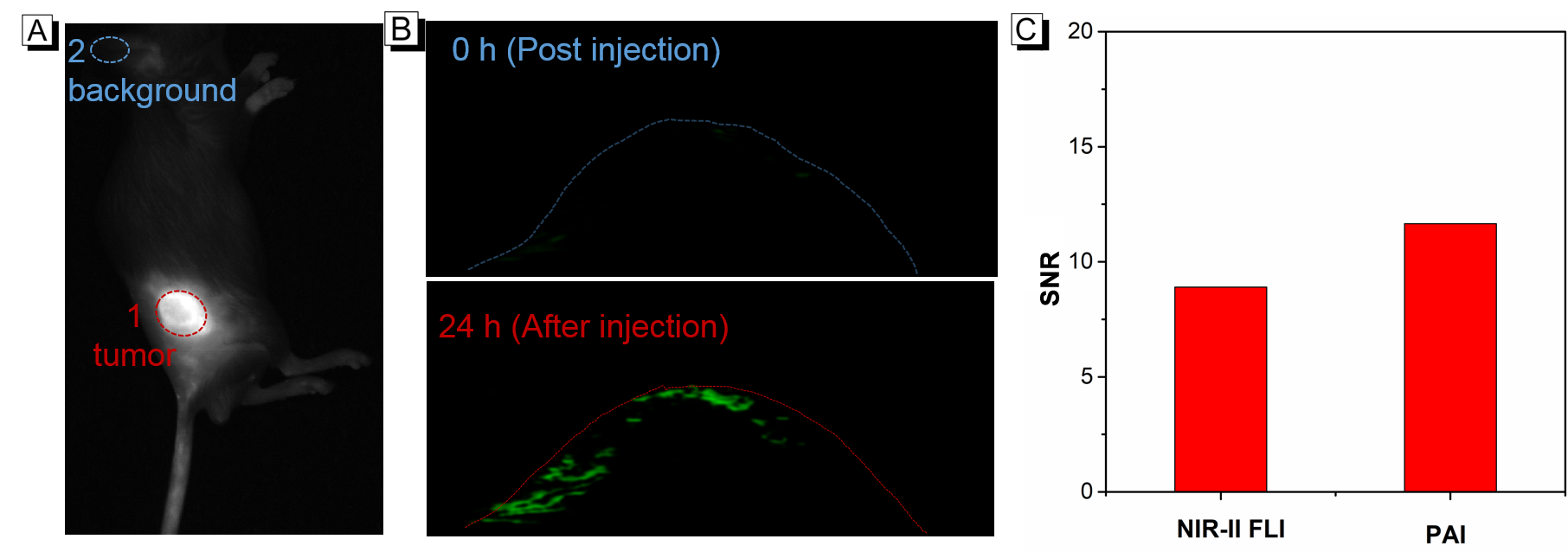


**Figure S22**. (A) NIR-II FLI and (B) PAI of tumor area at 24 h (down) after intravenous injection of TS NPs into 4T1-tumor-bearing mice and 0 h (up, post injection). (C) The SNR of NIR-II FLI and PAI of tumor area in 4T1-tumor-bearing mice.


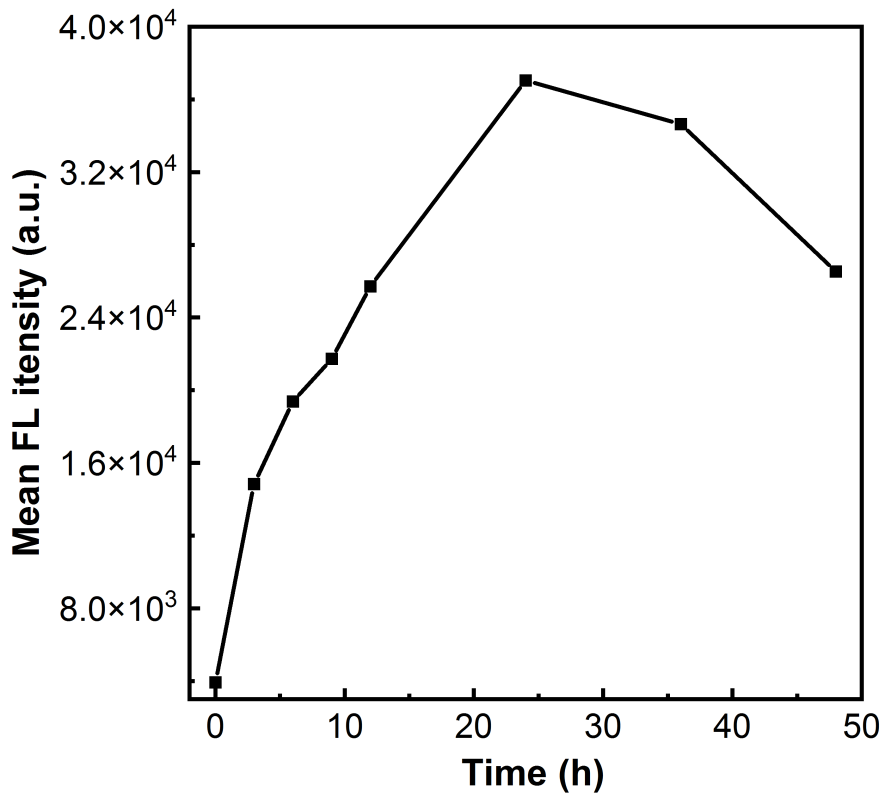


**Figure S23**. Curve plot of mean fluorescence intensity of tumor area at different time.


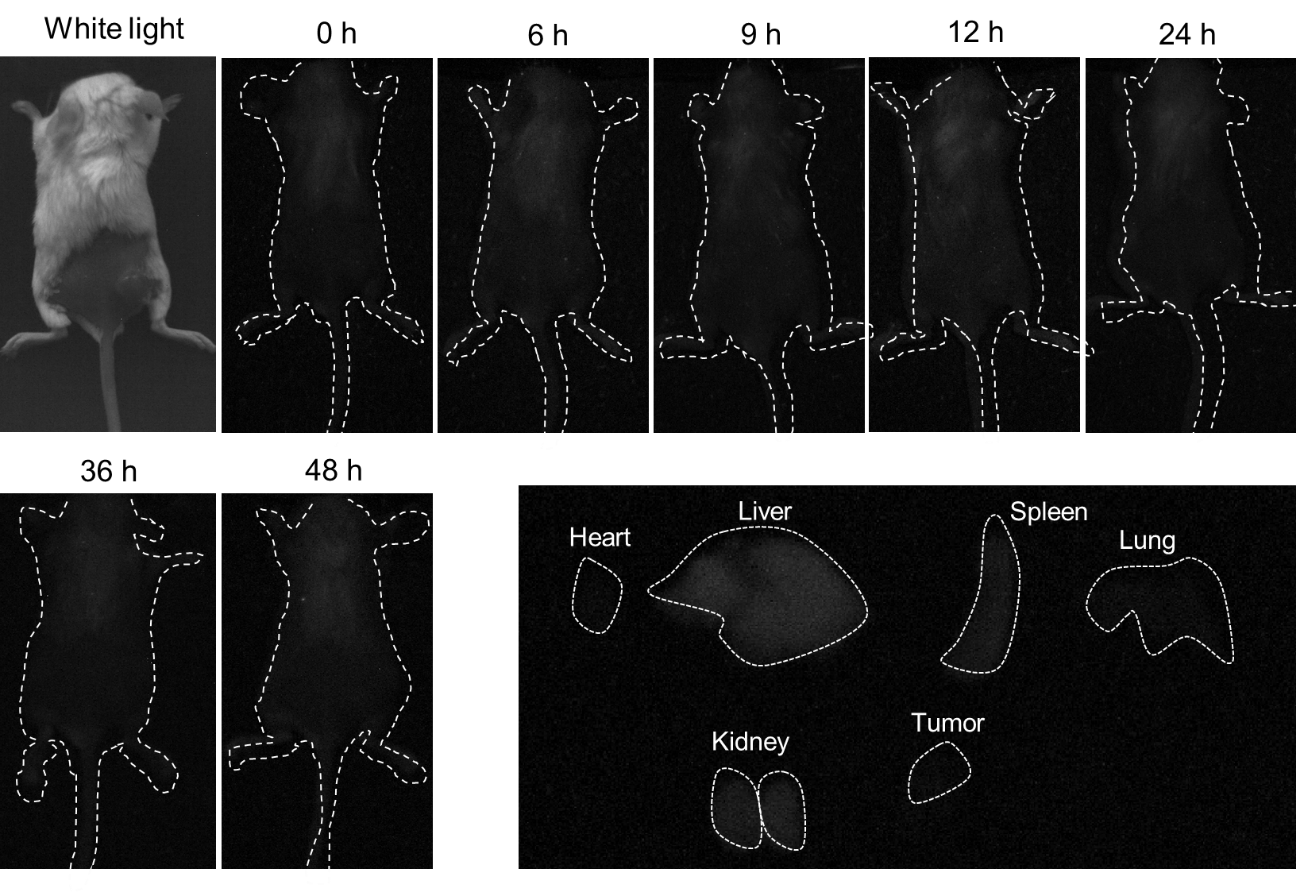


**Figure S24**. Time-dependent NIR-II fluorescence imaging for mice injected with PBS alone.


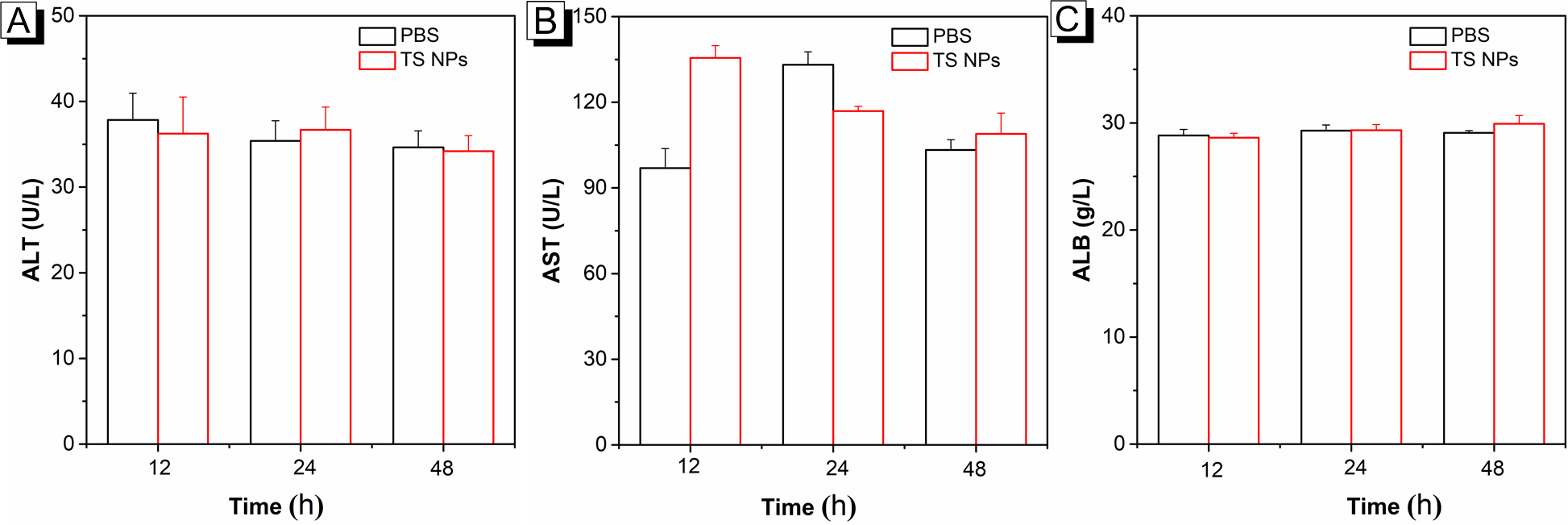


**Figure S25**. Blood biochemistry indexes of ALT, AST, and ALB for liver function evaluation.


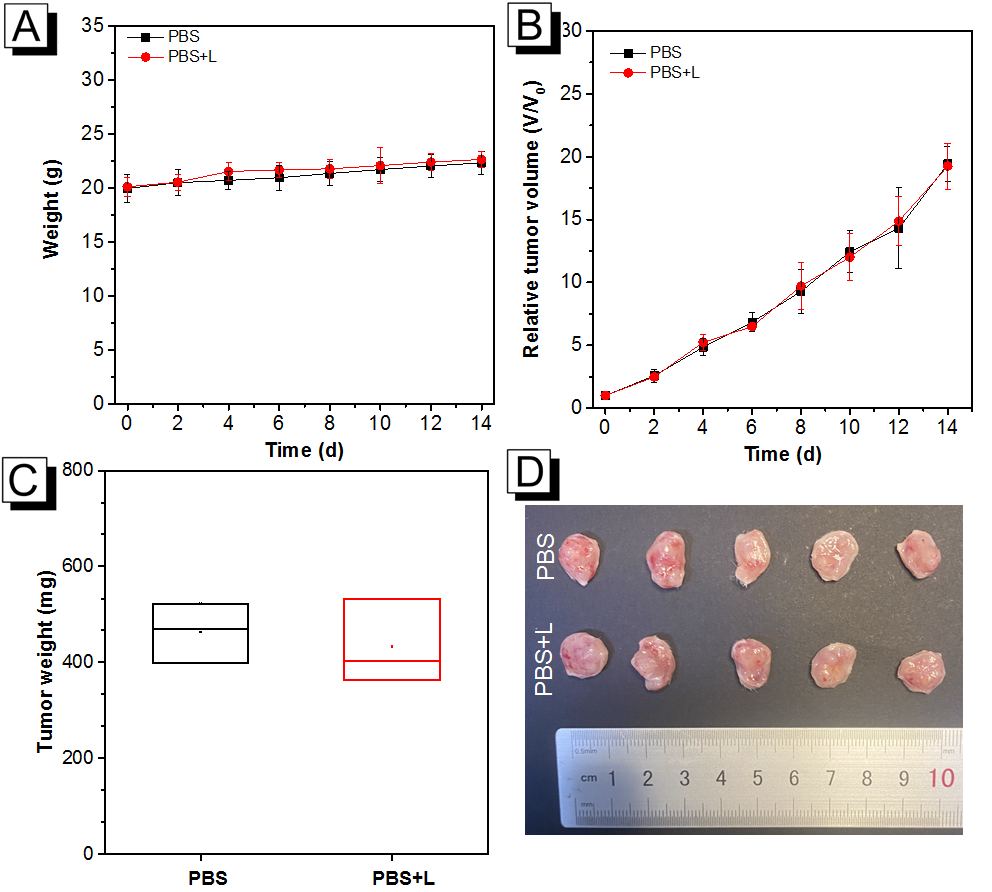


**Figure S26**. Growth curve of body weight (A) and tumor (B) of mice during the treatment process. (n = 5). Tumor weight (C) and tumor images (D) after 14 days’ treatment. (n = 5).


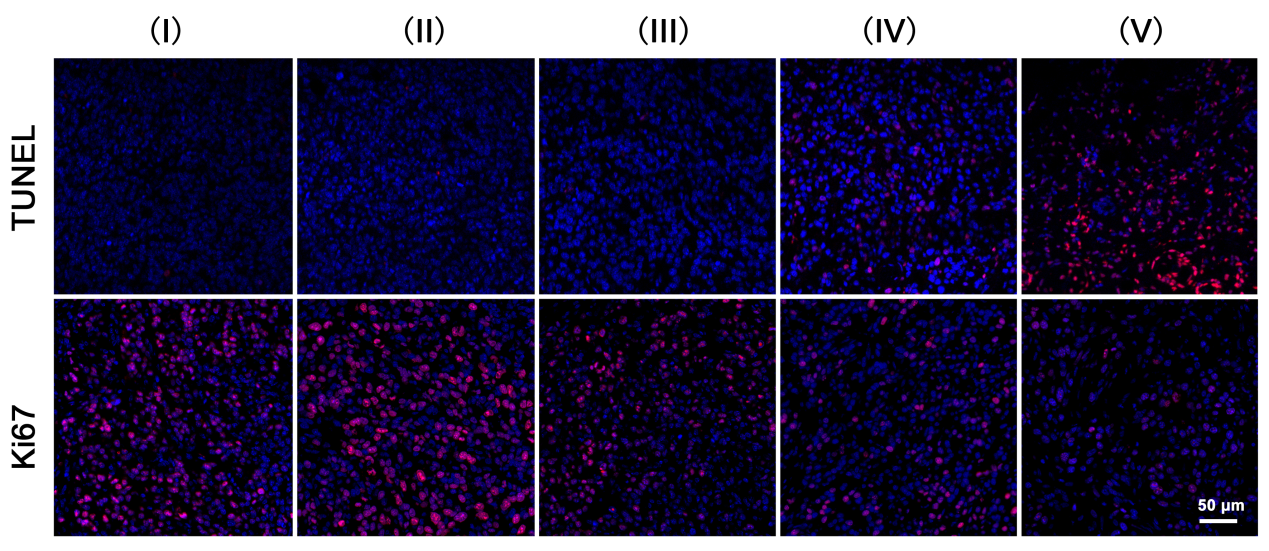


**Figure S27**. Histopathological view of the tumor tissue dissected from the mice in different groups after Ki67 and TUNEL staining.


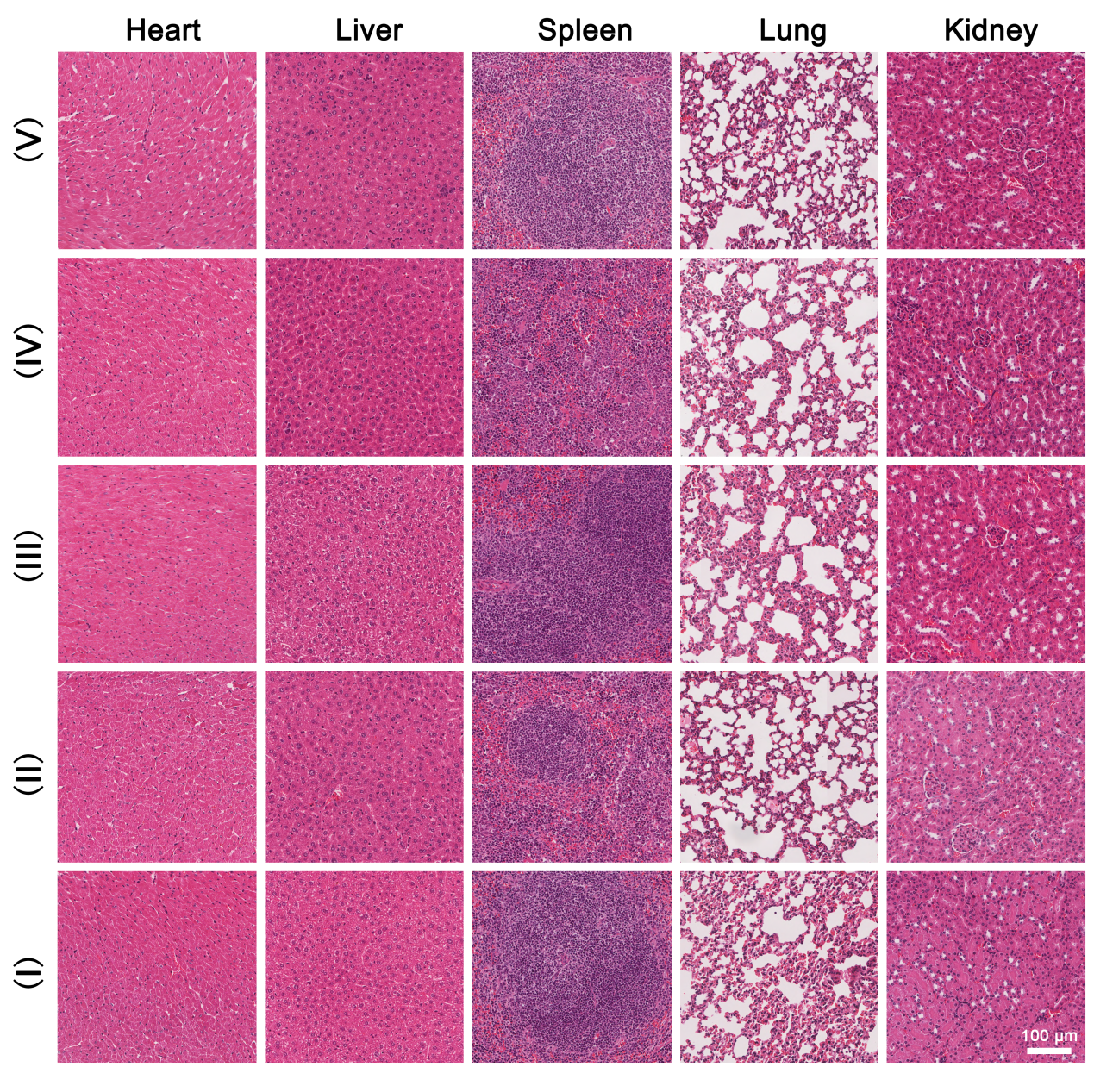


**Figure S28.** Hematoxylin and eosin (H&E) staining of the major organs sections dissected from mice in different groups after 14 days’ treatment.

References

[1] S. Hong, D.W. Zheng, C. Zhang, Q. X. Huang, S. X. Cheng, X. Z. Zhang. *Sci. Adv.* **2020**, *6*, eabb0020.
